# Integrated Multi-Omics Analysis of Cerebrospinal Fluid in Postoperative Delirium

**DOI:** 10.3390/biom14080924

**Published:** 2024-07-30

**Authors:** Bridget A. Tripp, Simon T. Dillon, Min Yuan, John M. Asara, Sarinnapha M. Vasunilashorn, Tamara G. Fong, Sharon K. Inouye, Long H. Ngo, Edward R. Marcantonio, Zhongcong Xie, Towia A. Libermann, Hasan H. Otu

**Affiliations:** 1 Department of Electrical and Computer Engineering, University of Nebraska-Lincoln, Lincoln, NE 68588, USA; 2 Genomics, Proteomics, Bioinformatics and Systems Biology Center, Beth Israel Deaconess Medical Center, Boston, MA 02215, USA; sdillon1@bidmc.harvard.edu (S.T.D.);; 3 Harvard Medical School, Boston, MA 02215, USA; jasara@bidmc.harvard.edu (J.M.A.); lngo@bidmc.harvard.edu (L.H.N.); zxie@mgh.harvard.edu (Z.X.); 4 Division of Signal Transduction and Mass Spectrometry Core, Beth Israel Deaconess Medical Center, Boston, MA 02115, USA; 5 Department of Medicine, Beth Israel Deaconess Medical Center, Boston, MA 02115, USA; 6 Harvard T.H. Chan School of Public Health, Boston, MA 02115, USA; 7 Department of Neurology, Beth Israel Deaconess Medical Center, Boston, MA 02115, USA; 8 Aging Brain Center, Marcus Institute for Aging Research, Hebrew SeniorLife, Boston, MA 02131, USA; 9 Department of Anesthesia, Critical Care and Pain Medicine, Massachusetts General Hospital, Boston, MA 02114, USA

**Keywords:** delirium, risk factors, multi-omics, lipidomics, proteomics, metabolomics

## Abstract

Preoperative risk biomarkers for delirium may aid in identifying high-risk patients and developing intervention therapies, which would minimize the health and economic burden of postoperative delirium. Previous studies have typically used single omics approaches to identify such biomarkers. Preoperative cerebrospinal fluid (CSF) from the Healthier Postoperative Recovery study of adults ≥ 63 years old undergoing elective major orthopedic surgery was used in a matched pair delirium case–no delirium control design. We performed metabolomics and lipidomics, which were combined with our previously reported proteomics results on the same samples. Differential expression, clustering, classification, and systems biology analyses were applied to individual and combined omics datasets. Probabilistic graph models were used to identify an integrated multi-omics interaction network, which included clusters of heterogeneous omics interactions among lipids, metabolites, and proteins. The combined multi-omics signature of 25 molecules attained an AUC of 0.96 [95% CI: 0.85–1.00], showing improvement over individual omics-based classification. We conclude that multi-omics integration of preoperative CSF identifies potential risk markers for delirium and generates new insights into the complex pathways associated with delirium. With future validation, this hypotheses-generating study may serve to build robust biomarkers for delirium and improve our understanding of its pathophysiology.

## 1. Introduction

Delirium is a condition characterized by an acute change and fluctuation in attention, thinking, and consciousness. Postoperative delirium affects 15–53% of older surgical patients and has been associated with extended hospitalization, significant postoperative complications, higher discharge rates to extended care facilities, and death [1,2]. In addition, delirium is associated with greater than USD 164 billion in annual U.S. healthcare expenditures, with USD 32.9 billion attributed to the postoperative setting alone [1,3]. 

Despite our growing understanding of the epidemiology of delirium, no laboratory test facilitates its diagnosis or mitigation. Instead, it is a wholly clinical diagnosis. Additionally, etiology and pathogenesis are poorly understood, challenging the discovery of biomarkers and clinical tests. Several biological models of pathogenesis have been proposed, including neuroinflammation, neurological aging, neuroendocrine stress, neurotransmitter dysfunction, oxidative stress, and dysregulation of the circadian rhythm [4,5].

Cerebrospinal fluid (CSF) provides brain protection, nourishment, and waste removal and is central for regulating nervous system functions. CSF is contained within the ventricles of the brain and the subarachnoid spaces of the skull and spine [6,7]. The choroid plexus (ChP) is a secretory tissue responsible for the primary source of CSF in the human brain. ChP secretes up to 500 mL of CSF daily, facilitating a total renewal of CSF four to five times a day [8]. This persistent turnover acts as the primary mechanism for transporting essential nutrients to the brain, assists in removing brain metabolic waste products and unnecessary molecules, and mediates the transport of circulating proteins such as cytokines and growth factors to different target cells in the brain [6]. The reduction in CSF turnover may result in an accumulation of unnecessary and toxic molecules that interfere with the neuronal functioning of the brain, as seen in aging and some neurodegenerative diseases [7]. CSF homeostasis is strictly regulated, and any variation in the molecular composition may be a useful diagnostic marker [9].

High-throughput technologies and omics approaches have opened the door to large-scale systems-level quantification of a diverse range of molecules within a biological system. These approaches can be applied to various biological matrices, such as plasma, serum, saliva, urine, and CSF. Omics is the complete cataloging of a molecular class within a system or phenotype. Omics approaches offer powerful tools for systems analysis, differentiating between two phenotypes, characterizing cellular changes in disease, and facilitating the identification of disease-specific markers.

Proteomics provides a comprehensive study of the proteome, the collection of proteins within a system [10]. The applications of proteomics approaches are many, ranging from discovering risk-and-disease markers to understanding disease pathogenesis. Proteins are involved in cellular processes, so perturbations in their expression may imply the underlying root of a diseased state. Metabolomics is a group of quantitative approaches to studying the collection of small metabolites in a system. Metabolites are often the end products and byproducts of biochemical processes in cells and are particularly sensitive to endogenous and exogenous stimuli [11]. Differences in their levels provide an efficient way to monitor and detect alterations in specific cellular pathways. Lipidomics—the high-throughput approach to cataloging lipid species within a system—is increasingly being applied in clinical research, offering new disease detection and prediction opportunities [12]. Lipids have numerous important roles in living organisms, especially in the central nervous system. Their concentrations can differentiate between a healthy versus a diseased state. They are essential components in the structure and function of the central nervous system. Lipid composition, transportation, and metabolism in neurons and astrocytes are integral to cellular health [13].

All three omics approaches, proteomics, metabolomics, and lipidomics, have previously been applied to the study of delirium pathogenesis and biomarker discovery using both CSF and plasma. Han et al. used an untargeted lipidomics approach to CSF collected preoperatively from elderly individuals undergoing hip fracture surgery. Their findings suggested phosphatidylethanolamine as a possible risk marker for delirium [14]. We previously used targeted metabolomics to examine plasma collected pre- and postoperatively in a matched case-control delirium study. These results showed perturbations in energy metabolism and that amino acid synthesis pathways may be associated with an increased risk of postoperative delirium [15]. Previous CSF metabolomics studies have supported the hypothesis that the genesis of delirium is rooted in an imbalance in aromatic amino acids [16]. In addition, the CSF proteome of delirium patients revealed evidence that inflammatory response is a key component of postoperative delirium [17]. We previously used mass-spectrometry-based plasma proteomics to define a preoperative delirium multi-protein signature that includes zinc-alpha-2-glycoprotein (AZGP1) and c-reactive protein (CRP) and a postoperative signature of interleukin-6 (IL-6), interleukin-2 (IL-2), and CRP [18]. We also used the high-throughput, aptamer-based SomaScan proteomics platform to identify high preoperative (PREOP) chitinase-3-like-protein-1 (CHI3L1/YKL-40) and high postoperative day 2 (POD2) IL-6 as risk and disease markers of postoperative delirium [19].

Previous studies have focused on a single omics approach. Biological systems consist of complex environments of interrelated pathways, and most disease phenotypes are driven by multiple perturbed components, which a single omics approach cannot sufficiently explain. Therefore, we applied a multi-omics approach to samples from a nested case-control study of matched postoperative delirium and no delirium samples from the Healthier Postoperative Recovery (HiPOR) cohort [20]. We conducted an exploratory, hypothesis-generating study, which, if validated in future work, can improve our understanding of delirium pathogenesis.

## 2. Materials and Methods

### 2.1. Human Subjects/Study Population

The HiPOR study protocol was approved by the Partners Human Research Committee (Boston, MA, USA), and all participants provided written informed consent. Eligible individuals were aged 63 years or older and were admitted for elective total knee and hip replacement using spinal anesthesia at the Massachusetts General Hospital [21,22]. Older adults with prior dementia were excluded based on patient or family report of dementia diagnosis, medical record review, or a baseline Mini-Mental State Examination (MMSE) score of less than 24 [23,24]. Additional exclusion criteria included severe visual or hearing impairment, stroke, and psychosis. For the current study, only participants enrolled between 2009 and 2016 with adequate banked CSF specimens were used.

### 2.2. Delirium Assessment and Matching

Delirium was assessed in HiPOR using the Confusion Assessment Method (CAM) based on the MMSE Mini-Mental State Examination (MMSE, purchased from Psychological Assessment Resources) for cognitive testing and the Delirium Symptom Interview (DSI) for patient symptom reporting [23,24,25,26]. CAM is a standardized, widely used, evidence-based tool that has been shown to have high sensitivity, specificity, and inter-rater reliability [27]. CAM diagnostic criteria for delirium require “acute change or fluctuating course in mental status” and “inattention” and either “disorganized thinking” or “altered level of consciousness”. Patients in the delirium group (DEL) met full CAM criteria on either postoperative day one or two. Patients with subsyndromal delirium were those who did not meet full CAM criteria but showed acute change or fluctuating course in mental status in addition to at least one of the remaining three CAM features. The remaining patients, without either full or subsyndromal delirium, were assigned to the control group (CNT). Patients with subsyndromal delirium were excluded from eligibility as either DEL or CNT.

DEL and CNT samples were matched on four patient criteria using the optimal match algorithm [28] to generate a nested, matched case-control study design that maximizes statistical power for discovery phase biomarker studies with small sample sizes [29]. Sex had to be an exact match, age within five years, year of surgery within five years, and baseline MMSE score within three points.

### 2.3. Collection and Processing of Cerebrospinal Fluid

Spinal anesthesia was administered to all HiPOR participants prior to surgery. While the anesthesia was being administered, 1 mL of CSF was collected using a spinal needle. The samples were stored in a −80 °C freezer following centrifugation at 1000× *g* for 10 min.

### 2.4. Proteomics

Protein quantification was performed using the SomaScan Assay Kit 1.3K, Cells and Tissue (item 900-00009) using serum diluent (SomaLogic, Boulder, CO, USA) as previously described [30,31]. The assay measured the levels of 1305 human proteins [32]. Raw data were processed through a quality control protocol, normalization, and calibration following the manufacturer’s instructions [33].

### 2.5. Targeted Metabolomics and Untargeted Lipidomics

Lipids and metabolites were extracted and measured using previously published untargeted and targeted methodologies, respectively [15,34]. For targeted metabolomics, a liquid chromatograph–mass spectrometry (LC-MS/MS) separation and metabolite identification (ID) was performed using the 5500 QTRAP hybrid triple quadrupole mass spectrometer (SCIEX) with fast positive/negative polarity switching. Rapid polarity switching allows a single run of sample to maximize metabolites identified [34]. Q1/Q3 multiple reaction monitoring (MRM) transitions were employed for definitive metabolite ID. The list of targeted molecules and their corresponding mass/charge (*m*/*z*) for the positive and negative ion modes (Supplementary Table S1) were used to link the molecular formula and PubChem Identifier (Supplementary Table S2). For the CSF-targeted metabolomics, 125 µL of each sample was extracted. In untargeted lipidomics, 40 µL per sample was processed for lipid isolation. Quality control measures applied to the platform can be found in Supplementary Table S8.

Data preprocessing followed the general workflow as previously described by Tripp and colleagues and outlined in Supplementary Figure S1 [15]. An analyte was considered “present” if measured in at least 50% of the samples within a phenotypic group (CNT, DEL). Signal drift was corrected using pooled quality control samples and a random forest signal correction (QC-RFSC) algorithm [35]. Signal imputation was performed using the k-nearest neighbor (knn) method [36]. Finally, metabolites and lipids were normalized to previously selected molecule-specific internal standards using the normalization method for metabolomics data using an optimal selection of multiple internal standards technique (NOMIS) (Table 1) [15,37].

As an artifact of the non-targeted lipidomics protocol, a lipid signal can be listed over multiple output lines. These lines represent distinct signal capture and were consolidated and summed before assessing if a lipid was “present”. The coefficient of variation was calculated for pooled quality control samples at each preprocessing step to evaluate the successful removal of technical noise introduced during data acquisition (Supplementary Table S3).

### 2.6. Statistical Analysis

Differential analysis was assessed using parametric and non-parametric statistical tests, namely, paired *t*-test, binomial test, and Wilcoxon signed-rank test, to account for the degree, direction, and rank of difference between delirium and control groups, respectively. To capture these different characteristics, metabolomics and lipidomics data were analyzed using all three tests. For each statistical test, the Benjamini–Hochberg (BH) procedure was applied to correct for multiple hypotheses testing [38]. The fold-change (FC) of an analyte was calculated by using one-step Tukey’s biweight algorithm on FC (tFC) values (DEL/CNT) for each matched pair [39]. This provides a robust estimation of the FC for each molecule that is unaffected by outliers. The FCs of the downregulated metabolites in the delirium group are indicated using the negative sign (e.g., an FC of −2 implies two-fold downregulation in the delirium group). Statistical analyses were performed using MATLAB (v.2021b, The MathWorks Inc., Natick, MA, USA).

### 2.7. Systems Biology

Metabolites (n = 51 of 219) and lipids (n = 26 of 161) with a nominal *p*-value < 0.05 in at least one statistical test were used as input for molecule-specific systems biology analysis (Table 2, Supplemental Table S4). Systems biology was performed using MetaboAnalyst (v3.0, www.metaboanalyst.ca, accessed on 2 June 2021), an online tool for analyzing metabolomics data, and LIpid Pathway Enrichment Analysis (LIPEA), a lipid-specific tool to identify altered pathways [40,41].

### 2.8. Multi-Omics Integration

Multi-omics integration and analysis were performed for the 15 matched pairs that had all three, metabolomics, lipidomics, and proteomics, data sets. In addition to the metabolites (n = 51) and lipids (n = 26) used for systems analysis, proteins previously identified and reported (n = 32) by Dillon and colleagues, which used the same experimental design, were also included. Signal, p-, and tFC values for all 109 molecules can be found in the Supplementary Data [31].

These 109 molecules were used as input into the multi-omics integration algorithm OBaNK: Omics Integration Using Bayesian Networks and External Knowledge (https://github.com/bridgettripp/OBaNK.git, accessed 25 September 2022) [42]. OBaNK learns the interaction structure of heterogeneous omics data through probabilistic graph modeling and further strengthens interactions through external knowledge from the Kyoto Encyclopedia of Genes and Genomes (KEGG), KEGG2Net, Recon3D, and SwissLipids [43,44,45,46]. Structure learning was performed on 1000 bootstrapped data sets, and the consensus network was obtained using model averaging [47]. The links in the consensus network were assigned a “strength value” based on their frequency of occurrence in the networks obtained by bootstrapping. These values were modified based on the incorporation of the external knowledge model as previously described [42]. Only links with modified strength values above the Scutari confidence threshold were included in the final network to denote significant interactions [48]. The output of OBaNK is a multi-omics interaction network where nodes represent molecules from different omics modalities and links represent interactions.

### 2.9. Machine Learning

Clustering of samples and molecules was performed using the Unweighted Paired Group Method with Arithmetic-mean (UPGMA) approach (a.k.a hierarchical clustering) with average linkage. Pearson’s correlation was used as the distance metric, and molecular expression data were standardized across samples prior to clustering [49]. The association of observed clusters with delirium was assessed using Fisher’s exact test.

Classification of samples was performed on data transformed using principal components analysis (PCA) [50]. Data were log-transformed and mean-centered for each feature prior to the application of PCA. Support vector machine classification (SVM) with linear, polynomial, and Gaussian kernels was used [51,52,53]. To further minimize the multi-omics signature and validate the contribution of integration of different omics, we used regularized logistic regression with elastic net [54]. Classification performance was assessed using leave-one-out cross-validation accuracy and area under the curve (AUC) of the receiver operating characteristic (ROC) curve [55,56]. All statistical and machine learning analyses were performed using MATLAB (v.2021b, TheMathWorks Inc., Natick, MA, USA).

## 3. Results

### 3.1. Sample Characteristics

The full cohort of 289 subjects was reduced to 103 (27 delirium, 76 non-delirium) prior to the match due to the exclusion of those (i) enrolled before 2009 (to limit sample degradation), (ii) without baseline cognition (MMSE), (iii) with low CSF volume (200 µL), and (iv) with subsyndromal delirium, missing data, or inability to determine delirium status. Applying our matching algorithm with the four matching factors (age within five years, exact sex, year of surgery within two years, MMSE score within three points) to the 103 subjects yielded 24 matched pairs (24 delirium cases, 24 non-delirium controls), which were run on the SomaScan platform for proteomic profiling of a total of 48 samples. Due to limited sample volumes and quality limitations, 18 and 16 delirium cases matched with no-delirium controls were processed for metabolomics and lipidomics, respectively. Fifteen matched pairs had all three omics signals measured (Supplementary Figure S2). Sample characteristics are shown in Table 3. Delirium and control groups showed no statistically or clinically important differences in the matched variables, and the different subgroups used for proteomics, metabolomics, lipidomics, and multi-omics were also similar in key variables. The difference in surgery date for all matched pairs was within two years, except for one matched pair used in metabolomics that had a five-year difference.

### 3.2. Molecules Altered in the Delirium Group at PREOP

Following data preprocessing, 100 percent of the metabolites and 98.8 percent of the lipids had a coefficient of variation of less than ten percent in the pooled quality control samples, showing successful removal of technical and experimental noise (Supplementary Table S3). A total of 109 molecules (proteins, lipids, metabolites) were statistically significantly altered in the delirium group at PREOP (*p* < 0.05) compared to the control group. After BH correction for multiple hypothesis testing, no proteins or lipids and only one metabolite (1,3-diphopshateglycerate) remained significant (Table 2), so we performed downstream analysis using molecules with significant nominal p-values. Among the 51 differentially expressed metabolites (Table 2a and Table 4), 44 (87%) were upregulated. However, we observed a reverse trend in the 26 lipids (Table 2b and Table 4), where 18 (69%) were downregulated, noted by a negative value under the fold change column, tFC. The previously reported 32 dysregulated proteins (Table 2b and Table 4) showed a more even distribution between up- and downregulated molecules, with 56 percent being upregulated.

Two of the three highest-fold change magnitudes within the metabolites with differential concentrations belonged to bacterial-derived analytes. Shikimate and indoleacrylic acid had the second and third greatest fold changes, with 2.25 and 2.02, respectively (Supplement Figure S3). Indoleacrylic acid is exclusively produced by the gut bacterial metabolism of tryptophan [57]. Shikimate is an intermediate within the shikimate pathway, a metabolic pathway observed in microorganisms and plants. S-adenosyl-L-homocysteine (SAH) had the fourth greatest change (tFC = 1.97). SAH is the metabolic precursor of homocysteine. The accumulation of homocysteine (hyperhomocysteinemia) in plasma reflects the functional status of three B vitamins (folate and vitamins B12 and B6) [58].

**Figure 1:**
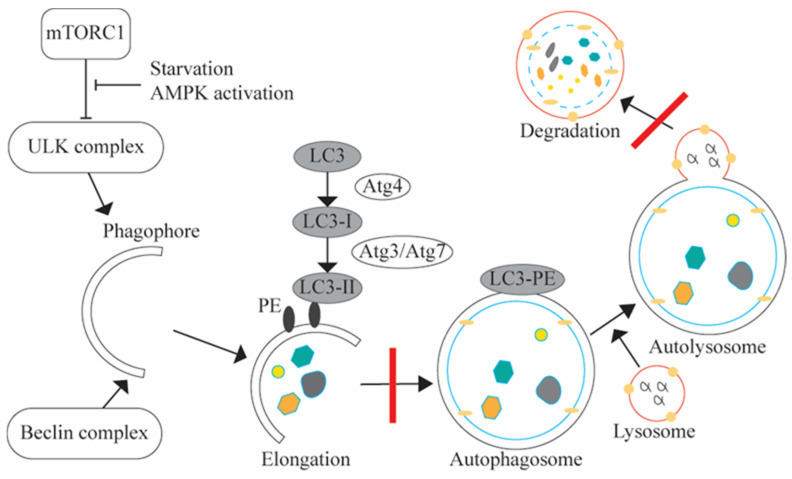
Generalized Autophagy Pathway. Red slashes denote the two positions within the pathway where the multi-omics results support dysregulation within the autophagy pathway. Four of the five phosphatidylethanolamines (PEs) recorded in our experiment were down in the delirium group. PE (black ovals) is critical to the elongation process in autophagy. Autophagy is a catabolic pathway that degrades cytosolic contents and is important for balancing energy stores in response to nutrient deprivation. It starts with the formation of the phagophore, which goes through an elongation process to form an autophagosome. A cytosolic microtubule-associated protein 1A/1B-light chain 3 (LC3) (denoted in grey) is conjugated to PE to form LC3-PE. Then, the mature autophagosome fuses with the lysosome. After fusion, lysosomal proteases, like cathepsin D (CTSD), degrade the contents of the autophagosome [59]. Our proteomics results showed a decline in CTSD in the delirium group, inferring a potential build-up of autophagosomes and, ultimately, the dysregulation of the autophagy pathway.

The 26 lipids with differential concentrations belonged to 11 lipid classes (Table 5). Multiple lipids belonged to one of four classes: phosphatidylcholines (PC), diacylglycerols, phosphatidylethanolamines (PE), and triacylglycerols. Sixteen of the twenty-six lipids were phospholipids, with PC and PE accounting for the majority. Of the sixteen phospholipids, eleven were down in the delirium group. PC species serve as a substrate for the synthesis of the neurotransmitter acetylcholine. Our results showed that four of the six lipids in the PC class were down preoperatively in patients developing postoperative delirium, three being in the top five with the highest tFC magnitude (Table 2b and Table 4). PE species are known for their essential role in the elongation of the phagophore to form the autophagosome in autophagy (Figure 1) [59]. Four of the five lipids that belonged to the PE class displayed a decrease in the delirium group (Table 5).

### 3.3. Pathway Enrichment Analysis of Lipids and Metabolites

**Figure 2:**
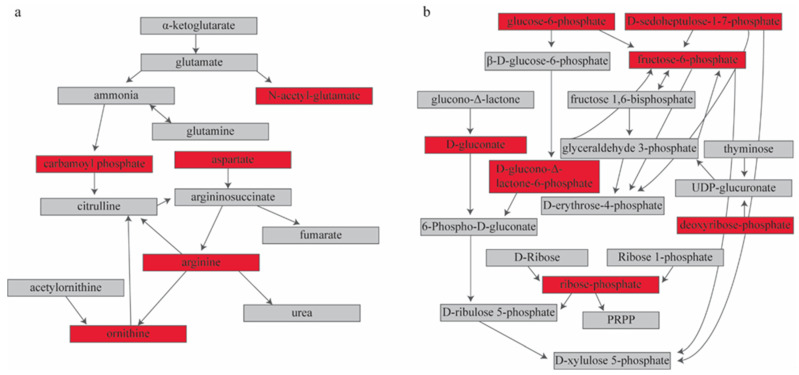
Pathway analysis of metabolites with significant differences in signal between the delirium and control groups. A red box indicates an input metabolite, and all are upregulated in delirium. The two most significantly enriched pathways are (**a**) arginine biosynthesis (FDR = 0.0011) and (**b**) pentose phosphate pathway (FDR = 0.00011). Supplementary Figure S4 contains the original pathway figures generated through MetaboAnalyst, including KEGG compound numbers.

MetaboAnalyst (v3.0, www.metaboanalyst.ca, accessed on 2 June 2021) returned six significantly enriched pathways with our input metabolites (Table 6) [40]. These pathways include the pentose phosphate pathway, arginine biosynthesis, glycolysis, alanine, aspartate, and glutamate metabolism, butanoate metabolism, and nicotinate and nicotinamide metabolism. Steps in and precursors to glycolysis are represented in this list of enriched pathways. The input metabolites contained within the two most significantly enriched pathways—the pentose phosphate pathway and arginine biosynthesis—were all upregulated and located in central positions within the pathways (Figure 2). This implies that both pathways function at a greater rate in the delirium group than in the matched controls before surgery. Studies have shown a significant increase in arginine is associated with cognitive decline [60,61]. The pentose phosphate pathway is an alternative pathway to glycolysis. Previous studies have shown dysregulation in alternative energy pathways in people with delirium [15].

Characterizing individual lipids and systems analysis approaches for lipids are nascent disciplines compared to other biomolecules. Evaluating the composition of lipids at the class level produces the most accurate snapshot of a system (Table 5). Our pathway enrichment analysis for the 26 significantly differentially expressed lipids using LIPEA identified seven pathways, including autophagy and glycerophospholipid metabolism (Supplementary Table S4) [41]. Although none of these enrichments showed statistical significance, the lipid pathway association provides important descriptive information that may help guide future pathophysiologic investigations.

### 3.4. Signature Prediction of Delirium with Machine Learning

**Figure 3:**
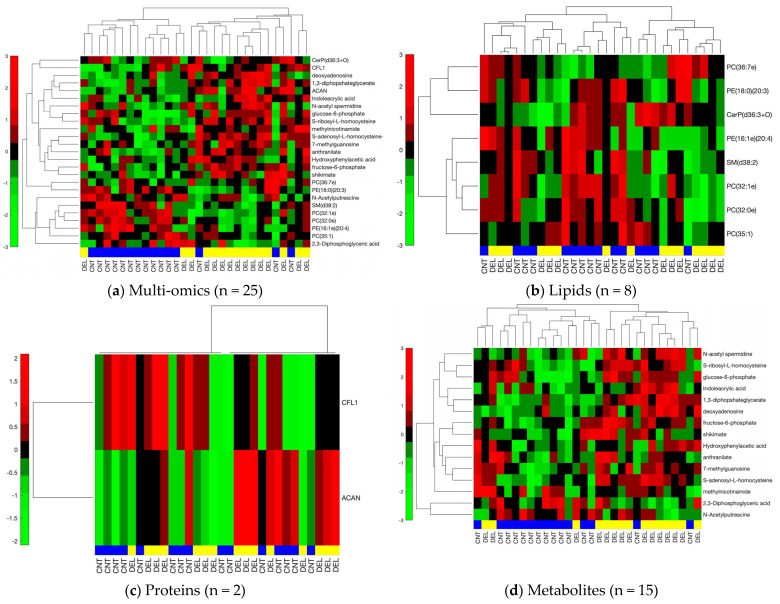
Hierarchical clustering for lipid, metabolite, protein, and combined omics signatures. The blue and yellow horizontal bars denote control (CNT) and delirium (DEL) samples, respectively. Red and green represent up- and downregulation in the delirium group, respectively. The vertical side bar represents the range of row-normalized (zero-mean, unit-variance) signal values and corresponding color codes. (**a**) Combined multi-omics signature (25 molecules), (**b**) Lipidomic signature (8 lipids), (**c**) Proteomic signature (2 proteins), (**d**) Metabolomic signature (15 metabolites).

We further constricted the differentially abundant molecules by using a |tFC| > 1.5 cut-off to utilize high fold changes and to generate a reasonably-sized multi-omics list to be used as a predictive signature and to be analyzed in an interaction network setting. This resulted in two proteins, fifteen metabolites, and eight lipids that were analyzed separately and together for their clustering and classification performance. Figure 3 shows the hierarchical clustering results for the separate and combined omics profiles. We observed thirteen, six, and eleven out of thirty incorrectly clustered samples when individual proteomic, metabolomic, and lipidomic signatures were used, respectively. However, we only had five out of thirty samples incorrectly clustered when all three omics signatures were combined to obtain a multi-omics signature. Clusters observed using metabolomics and multi-omics signatures rendered significant association with delirium status (*p* < 0.0025 and *p* < 0.0007, respectively), while proteomic and lipidomic signatures did not provide clusters significantly associated with delirium. These results suggest an improvement in clustering when an integrated multi-omics signature is used.

**Figure 4:**
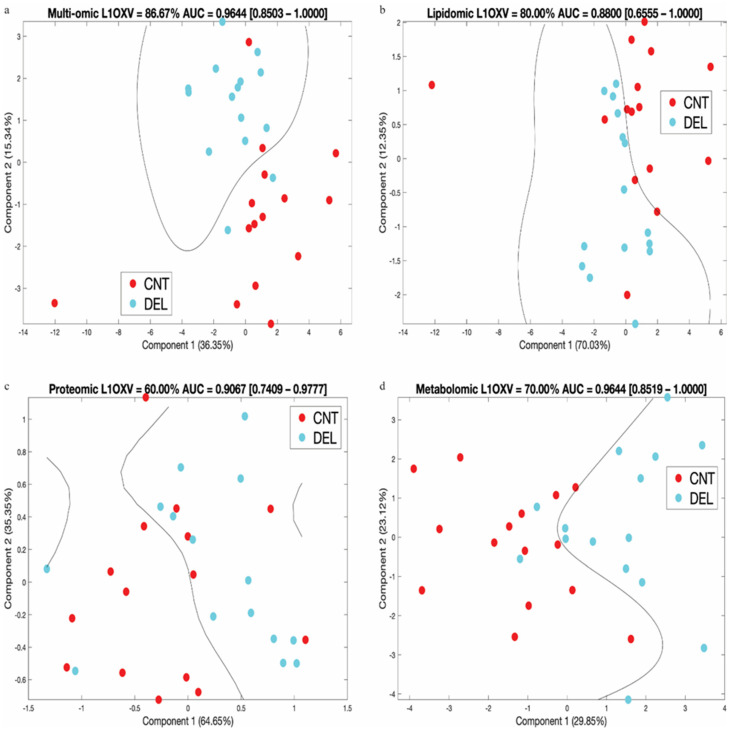
For each signature, leave-one-out cross-validation (L1OXV) accuracy and area under the curve (AUC) of the receiver operating characteristic (ROC) curve are noted. In brackets, we note the 95% confidence interval for the AUC values. (**a**) Combined multi-omics signature (25 molecules), (**b**) Lipidomic signature (8 lipids), (**c**) Proteomic signature (2 proteins), (**d**) Metabolomic signature (15 metabolites). Percent values along the axes represent the percent of variation in data explained by the respective principal component.

In Figure 4, we show the SVM prediction results on data transformed using PCA analysis. We obtained the lowest cross-validation accuracy with the proteomic signature (60%) that improved for the metabolomic (70%) and lipidomic signatures (80%), peaking for the combined multi-omics signature (87%). The AUC values for the lipidomic and proteomic signatures were 0.88 and 0.91, respectively, while the combined multi-omics and metabolomics signature attained a higher AUC (0.96) (see Supplementary Figure S5 for the ROC curves). As shown in Figure 4, the distance of the samples to the decision line, i.e., the margin in SVM classification, was wider in the multi-omics signature compared to individual omics signatures, indicating a more robust classification model. The multi-omics signature stood out as the best overall performing signature when all of the clustering, cross-validation, and AUC results were considered.

**Figure 5:**
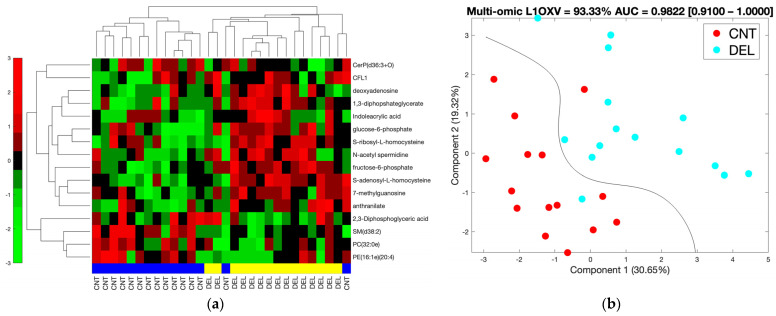
(**a**) Hierarchical clustering and (**b**) PCA+SVM plot using the refined multi-omics signature with 16 molecules (11 metabolites, 5 lipids, and 1 protein). The molecules that ended up in the majority of leave-one-out-cross-validation models in regularized logistic regression with elastic net analysis constitute the refined list of 16. The input list for the regression analysis was the 25 molecules (15 metabolites, 8 lipids, 2 proteins) that were significantly associated with delirium (*p* < 0.05) and showed high fold change (|tFC| > 1/5).

We analyzed individual and combined omics with and without a fold change cut-off of 1.5 using regularized logistic regression with elastic net. We used model coefficients that corresponded to the minimum expected deviance based on 3-fold cross-validation. A sample deviance plot is shown in Supplementary Figure S6. Summarized in Supplementary Table S6, the best performing set was the 25-molecule multi-omics data (8 lipids, 15 metabolites, 2 proteins) that represented a significant association with high fold change and was used in the analysis shown in Figure 3 and Figure 4. We further analyzed the 25-molecule results by identifying the number of times a feature was included in the final model out of 30 leave-one-out model generations. We identified 16 molecules: 4 lipids, 11 metabolites, and 1 protein that were involved in the majority of the models. Using these 16 molecules, we performed hierarchical clustering, which resulted in two samples that were wrongly clustered, and our PCA+SVM workflow, which resulted in 93.33% L1OXV accuracy and 0.9822 AUC of ROC with a 95% confidence interval of [0.91–1.00] (Figure 5). We believe our results underline the value of integrating multiple omics for classification purposes, as the final 16-molecule set obtained through this parsimonious approach included all three omics and yielded the highest accuracy and AUC values.

### 3.5. Multi-Omics Integration

**Figure 6:**
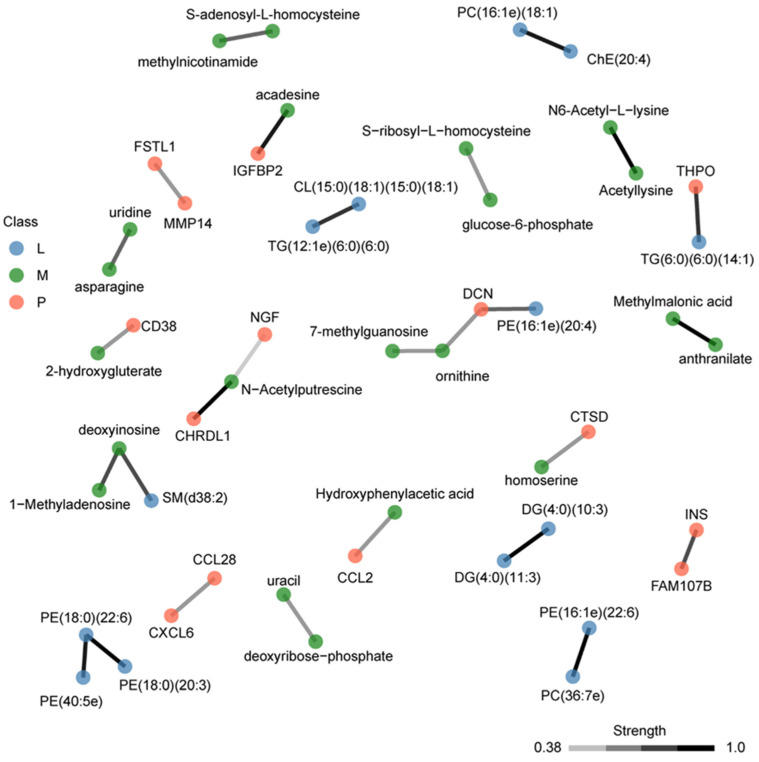
Multi-omics integration using OBaNK. A total of 109 molecules were used as input (lipids = 26, metabolites = 51, proteins = 32) (Table 2, Supplemental Table S4). Nodes are colored to represent the molecule type (blue = lipids; green = metabolites; orange = proteins). Edges represent the significant interactions (strength) between molecules. Edges are linearly color-coded to represent the interaction confidence [0.38–1.0], with black representing the highest confidence.

We used OBaNK to integrate the three different omics experiments using the 109 multi-omics molecules with a *p*-value < 0.05. OBaNK takes as input biological data and learns a molecular interaction network. The links are learned directly from the multi-omics input data using a Bayesian network structure learning algorithm, and connections are supplemented with external interaction knowledge [42]. This method improves the traditional interaction network approaches, which typically do not incorporate existing knowledge and are limited by single omics where heterogeneous molecular interactions, i.e., interactions between different molecule types, e.g., proteins and metabolites, are not accounted for, failing to represent a systems-level view. Connecting the three functional omics layers using OBaNK identified a multi-omics interaction network with 22 subnetworks of at least two or more molecules (Figure 6). Edges were linearly color-coded to represent the interaction confidence; only those links with significant confidence were included, with black representing the highest confidence. One of the subnetworks shown in Figure 6 was a tri-omics (lipidomics, metabolomics, and proteomics) cluster. Eight, including the tri-omics group, involved interactions of two or more types of molecules. Two heterogenous clusters represent molecules in histone methylation and chromatin accessibility, and redox homeostasis.

## 4. Discussion

In this proof-of-concept exploratory study, we applied targeted metabolomics, proteomics, and untargeted lipidomics approaches to preoperative CSF to quantify potential molecules associated with the risk of postoperative delirium after orthopedic surgery under spinal anesthesia and integrated these results using systems biology and machine learning. While individual omics performed well in classifying patients who went on to develop delirium vs. those who did not, combining lipidomics, metabolomics, and proteomics improved clustering accuracy and discriminatory abilities. Systems analysis of different omics may present a fuller picture when collectively considered, as observed in improved classification accuracy and AUC performance, and our probabilistic graph model supplemented with external knowledge elucidated interactions between different molecule types. Integrated multi-omics analysis has the potential to provide a better picture of the underlying mechanisms not attainable with a single omics approach. It should be noted that our proof-of-concept study is intended for hypothesis generation to guide future exploration and was not validated using an external cohort.

CSF metabolomics analysis at PREOP in the HiPOR study identified 51 significant delirium-associated metabolites. We previously analyzed the metabolome associations with postoperative delirium in plasma at PREOP and POD2 for a different elective, primarily orthopedic surgery cohort, the Successful Aging after Elective Surgery (SAGES) study and examined whether there existed any overlap between the HiPOR CSF and the SAGES plasma metabolome data [15]. We observed that six metabolites were in common between HiPOR CSF PREOP and SAGES plasma PREOP delirium-associated metabolites, and nine metabolites were in common between HiPOR CSF PREOP and SAGES plasma POD2 delirium-associated metabolites, with two of them, S-ribosyl-L-homocysteine and D-gluconate, common to all three metabolomics analyses (Supplementary Table S7). Interestingly, the fold change directionality between CSF and plasma was more congruent between CSF PREOP and plasma POD2 than plasma PREOP. Specifically, among the six metabolites shared between CSF PREOP and plasma PREOP only D-gluconate and 3-hydroxybuterate had the same directionality. In contrast, all nine metabolites, D-gluconate, S-ribosyl-L-homocysteine, S-adenosyl-L-homocysteine, 1-methyladenosine, acetyllysine, SBP, D-sedoheptulose-1-7-phosphate, deoxyinosine, and D-glucono-delta-lactone-6-phosphate, common to CSF PREOP and plasma POD2 had consistent directionality, all being increased in delirium cases. Thus, despite CSF and plasma being collected from different cohorts, there is nominal overlap between metabolites associated with delirium across both studies, even though this is significant only for CSF at PREOP with plasma at POD2 (*p* < 0.01) [62].

Our previous POD2 plasma metabolomics analysis suggested that the dysregulated expression of kynurenic acid, which is a degradation product of tryptophan, could be associated with delirium [15]. While in this study, we did not observe significant alterations in kynurenic acid in CSF, a significant increase in kynurenine, a tryptophan degradation product upstream of kynurenic acid, was prominent. An increasing number of publications link the regulation of kynurenine by inflammatory cytokines to neurological diseases [63]. Enhanced expression of kynurenine is linked to inflammatory states and in response to immune system activation, and upregulated plasma levels of kynurenine have been shown to be an independent predictor of greater duration of delirium in the ICU and of mortality and neurological outcomes in cardiac arrest patients [64]. Neuroinflammation-mediated kynurenine upregulation is found in serum, plasma, CSF, and brain tissue in a range of neurodegenerative disorders, e.g., Alzheimer’s disease, multiple sclerosis, and Parkinson’s disease [63,65,66]. We also observed an elevated level of N6-Acetyl-lysine. Both metabolites have been identified as upregulated in a whole blood metabolomics analysis of patients with dementia [67]. N6-Acetyl-lysine may be associated with the accumulation of acetylated Tau in Alzheimer’s disease [68,69].

Over the last two decades, research has supported a bidirectional communication system between the central nervous system and the microbial community in the gastrointestinal tract, referred to as the gut–brain axis [70]. Two of the metabolites with the highest positive fold change difference between delirium and healthy controls belong to a group of bacterial-derived analytes: shikimate and indoleacrylic acid. Indoleacrylic acid is a product of tryptophan metabolism by gut bacterial communities [57]. The upregulation observed in the delirium group could imply greater metabolism of dietary tryptophan. Shikimate is an intermediate within the shikimate pathway, a metabolic pathway observed in microorganisms and plants. This pathway synthesizes folates and the aromatic acids phenylalanine, tryptophan, and tyrosine [71]. As part of the gut–brain axis, signals from the brain control functions in the gut, while the brain and gut communicate via a battery of physiological channels, including molecules synthesized by the gut microbiota [70]. Studies have shown that the gut microbiota can modulate the two major tryptophan metabolism pathways: serotonin and kynurenine. Dysregulations in these pathways lead to disequilibrium in cognitively important metabolites and neurotransmitters [70,72]. Recent studies focusing on the gut microbiota composition in older patients found that microbiota associated with inflammation pathways (e.g., *Parabacteroides distasonis* and *Prevotella*) and neurotransmitter modulation were commonly higher in those who experienced delirium [73,74,75]. Future studies may use the suggested system from the present study to determine the associations between gut microbiota and other potential biomarkers of postoperative delirium in patients.

Enrichment analysis revealed that amino acid biosynthesis (arginine biosynthesis) and metabolism (alanine, aspartate, and glutamate metabolism) possibly play a role in delirium. These findings are consistent with amino acid metabolism alterations seen in our prior plasma metabolomics study and another study of preoperative CSF in elderly patients with hip fracture surgery [14,15]. They have also been associated with Alzheimer’s disease and dementia [76]. We identified increased expression of S-adenosyl-L-homocysteine (SAH) in the CSF at PREOP and previously in plasma at POD2 [15]. SAH plays an apparent role in cognitive functions and is increased in cognitively impaired individuals in CSF and plasma [77]. SAH is the metabolic precursor of homocysteine. Hyperhomocysteinemia has been identified as a risk marker for cognitive decline, delirium, dementia, and Alzheimer’s disease in older adults [78,79]. Levels of SAH also correlate with p-Tau181 [77]. Higher levels of SAH also correlate with an increased risk of death in a prospective study of an older population [78].

Of the 26 lipids we identified, two downregulated phosphoethanolamines (PE (16:1e) (22:6), PE (18:0) (22:6)) involved in glycerophospholipid metabolism had previously been identified among preoperative CSF lipids decreased in delirium in elderly hip fracture patients [14]. We observed lipid membership in three pathways: autophagy, ferroptosis, and retrograde endocannabinoid signaling, which have previously been implicated in neurodegenerative diseases. Autophagy is a catabolic process resulting in the degradation of cytoplasmic contents generally activated by nutrient deprivation [80]. Ferroptosis is a programmed cell death that occurs when glutathione-dependent antioxidant defenses fail. It has been linked to neurodegenerative diseases, including Alzheimer’s disease [81]. Retrograde endocannabinoid signaling performs neuromodulation in the brain, and endocannabinoids can regulate several neural functions [82]. All three pathways displayed lower availability of lipid species from the phosphatidylethanolamine (PE) class in the delirium group, thus implying a potential perturbation in phospholipid biosynthesis. Wallace et al. identified a strong inverse relationship between inflammatory markers, CRP, TNF-α, resistin, and MCP-1, and lipids in the lysophosphatidylcholine (LPC), phosphatidylcholine (PC) and phosphatidylethanolamine (PE) classes [83]. We observed eight significantly downregulated lipids from the PC and PE classes in delirium. This might imply an upregulation in the associated inflammatory markers. An increase in CRP and TNF-α in delirium has been observed [84,85,86].

Multi-omics integration using OBaNK identified several interactions between metabolites, lipids, and proteins. To evaluate whether these predicted potential interactions include known biological relationships we looked in more detail at several of these interactions. For example, the predicted interaction between CD38 and 2-hydroxygluterate (2-HG) (Figure 6) is strongly supported by findings in T lymphocytes where CD38 regulates the intracellular levels of 2-HG, which plays a pivotal role in histone methylation and chromatin accessibility [87]. The NAD+ degrading enzyme CD38 levels increase during aging, may regulate Alzheimer’s disease pathology, and play a role in neuroinflammation [88,89].

Another interaction cluster included biotinyl PE (25:0), D-glucono-delta-lactone-6-phosphate, guanine, and PPIF. The metabolite D-glucono-delta-lactone-6-phosphate is an intermediate in the pentose phosphate pathway. When functioning properly, the pentose phosphate pathway is neuroprotective. Microglial and immune cells generate high concentrations of glucose-6-phosphate dehydrogenase, a rate-limiting enzyme for the pentose phosphate pathway [90]. When microglial cells have excessive activation of glucose-6-phosphate dehydrogenase, this can lead to an overabundance of reactive oxygen species [91]. PPIF is a component of the mitochondrial permeability transition pore and is involved in mitochondrial apoptosis. PPIF induces a conformation change in the mitochondria, increasing reactive oxygen species levels [92]. The increased levels of reactive oxygen species ultimately lead to mitochondrial apoptosis. This heterogeneous cluster, therefore, appears to be linked to reactive oxygen species. Previous studies have suggested dysregulation in redox homeostasis can lead to postoperative delirium [4].

Individual omics analysis on its own can guide future experiments and elucidate dysregulations between case and control cohorts. However, most conditions are rooted in more complex etiologies and the complete picture may be missed when single omics data are analyzed. For example, based on our lipidomics results only, dysregulation in autophagy is weakly linked to the delirium group. However, when the analysis is expanded to the proteomics and metabolomics results, a more complete picture emerges. There is evidence that a hyperhomocysteinemic state, which is implied by the accumulation of SAH in our metabolomics results, impairs autophagy, resulting in cellular injury in a murine brain model [93]. Increasing evidence suggests that a hyperhomocysteinemic state is associated with brain microvasculopathy [94,95,96]. Specifically, an elevated homocysteine level has been reported to be associated with cerebral microangiopathy but not with cardioembolic or macroangiopathic mechanisms [97]. Brain microvasculopathy is also associated with delirium, and it is plausible that a hyperhomocysteinemic state may increase the risk of postoperative delirium via potentiating brain microvasculopathy [98,99]. Future studies to test this hypothesis are warranted. In our lipidomics results, we observe reduced availability of phosphatidylethanolamine, a critical component in the formation of the autophagosome used in macroautophagy (Figure 1). Under normal conditions, the lysosome fuses with the mature autophagosome, and a lysosomal protease like cathepsin D (CTSD) degrades the contents [59]. Our proteomics results showed a decline in CTSD in the delirium group. All three omics results point to a possible dysregulation in autophagy.

Despite our strengths in multi-omics integration, experimental design, established cohort, developed workflow, and data analysis methods, several limitations of this study are of note. The sample size was limited in all three omics, leading to insufficient statistical power to yield significant BH-corrected molecules. Although not being corrected for multiple hypothesis testing, the identified molecule list validated some of the molecules in previously published omics studies and showed highly successful discriminatory power for clustering, classification, and prediction. Furthermore, the systems and pathway analysis demonstrated the relevance of the identified molecule list as they were coherent with each other and consistent with the existing biological and clinical literature. However, it should be noted that our proof-of-concept study is hypothesis generating as it is an exploratory study, lacking an independent validation cohort. Second, lipidomics is a newer omics methodology, so analytical tools are limited. Additionally, nomenclature between analytical tools is not standardized, so findings are limited to only those lipids properly labeled for a specific approach. This results in lipid presence often being under reported. Finally, targeted metabolomics and proteomics are restricted by the detection of the predefined molecules established by the specific protocol; however, our analytical platforms do provide some of the broadest samplings available on a targeted platform. This may result in the omission of relevant molecules involved in delirium.

## 5. Conclusions

In summary, we report, to our knowledge, the first multi-omics analysis of preoperative CSF for delirium risk biomarkers, combining proteomics, metabolomics, and lipidomics. We demonstrate that such a multi-omics approach may generate multi-analyte prediction models with improved performance as compared to single omics-based models. Moreover, our multi-omics OBaNK analytic strategy enables us to explore potential interactions between different omics data that help to reveal plausible pathophysiological mechanisms. Despite the relatively small sample size, our computational analysis produced promising associations, clustering, prediction, and interaction results, and the use of metabolomics and lipidomics, in addition to proteomics, provided novel insights that would otherwise not be possible with single-omics analysis. Although exploratory at this level, if validated in future work, this multi-omics signature can serve to both increase the predictive power for postoperative delirium and improve our understanding of delirium pathogenesis. Further studies in independent cohorts are warranted to confirm and extend the findings from this study.

## Figures and Tables

**Table 1:** Metabolomics Internal Standards (ISs). Optimal internal standards were selected using previously published protocols [15]. All non-IS molecules were normalized to their respective ISs using NOMIS [35].

Lipids	Metabolites
LPE (17:1)	L-Tryptophan_D3_pos
PE (15:0) (18:1)	DL-Valine_D8_pos
PG (15:0) (18:1)	DL-Alanine_D3_pos
PI (15:0) (18:1)	DL-Alanine_D3_pos

**Table 2:** Metabolites (a), lipids (b), and proteins (c) with a significant difference (nominal *p*-value < 0.05) between delirium and control groups in at least one statistical test. The lowest nominal *p*-values are listed. Tukey fold changes (tFC) of the downregulated lipids in the delirium group are indicated using a negative sign. Molecules are ordered by tFC.

**(a)**							
**Metabolite**	***p*-Value**	**BH *p*-Value**	**tFC**	**Metabolite**	***p*-Value**	**BH *p*-Value**	**tFC**
**deoxyadenosine**	0.0008	0.0557	2.46	ornithine	0.0481	0.2451	1.27
**shikimate ^1^**	0.0154	0.2114	2.25	D-gluconate	0.0481	0.2451	1.26
**indoleacrylic acid ^1^**	0.0302	0.2451	2.02	kynurenine	0.0334	0.2451	1.25
**S-adenosyl-L-homocysteine**	0.0005	0.0540	1.97	deoxyinosine	0.0481	0.2451	1.25
**methylnicotinamide**	0.0481	0.2451	1.84	acetyllysine	0.0348	0.2451	1.25
**anthranilate ^1^**	0.0210	0.2451	1.81	N-acetyl-glutamate	0.0007	0.0632	1.23
**1,3-diphopshateglycerate**	0.0002	0.0418	1.80	D-sedoheptulose-1-7-phosphate ^1^	0.0160	0.2180	1.25
**N-acetyl spermidine**	0.0077	0.1290	1.70	aspartate	0.0481	0.2451	1.17
**glucose-6-phosphate**	0.0050	0.1175	1.66	2-hydroxygluterate	0.0067	0.1175	1.16
**hydroxyphenylacetic acid ^1^**	0.0481	0.2451	1.64	Succinate ^1^	0.0481	0.2451	1.14
**7-methylguanosine**	0.0035	0.1175	1.62	phenylpropiolic acid	0.0023	0.1023	1.13
**S-ribosyl-L-homocysteine ^1^**	0.0059	0.1175	1.58	2-oxo-4-methylthiobutanoate	0.0038	0.1376	1.13
**fructose-6-phosphate**	0.0052	0.1175	1.56	deoxyribose-phosphate	0.0342	0.3151	1.13
**SBP**	0.0481	0.2451	1.49	arginine	0.0481	0.2451	1.11
**D-glucono-delta-lactone-6-phosphate**	0.0481	0.2451	1.48	methylmalonic acid	0.0033	0.1175	1.10
**3-methylphenylacetic acid ^1^**	0.0077	0.1462	1.45	asparagine	0.0154	0.2114	1.09
**nicotinamide riboside**	0.0431	0.1462	1.45	dGMP	0.0290	0.2857	1.01
**uracil**	0.0070	0.1175	1.41	trehalose-sucrose	0.0389	0.2451	0.90
**1-Methyladenosine ^1^**	0.0135	0.1847	1.45	3-hydroxybuterate	0.0485	0.3365	−0.81
**guanine**	0.0481	0.2451	1.39	dTTP	0.0432	0.3269	−1.13
**uridine**	0.0054	0.1175	1.37	alanine	0.0481	0.2451	−1.15
**carbamoyl phosphate**	0.0240	0.2749	1.33	dihydroxy-acetone-phosphate	0.0481	0.2451	−1.43
**homoserine**	0.0481	0.2451	1.32	2-deoxyglucose-6-phosphate	0.0481	0.2451	−1.46
**ribose-phosphate**	0.0328	0.2451	1.31	N-Acetylputrescine	0.0429	0.3151	−1.54
**acadesine**	0.0481	0.2451	1.30	2,3-Diphosphoglyceric acid	0.0154	0.2114	−1.67
**N6-Acetyl-L-lysine**	0.0226	0.2451	1.29				
**(b)**							
**Lipid**	***p*-Value**	**BH *p*-Value**	**tFC**	**Lipid**	**p-value**	**BH p-value**	**tFC**
**PC (36:7e)**	0.038	0.269	1.52	DG (18:3e)	0.038	0.269	−1.33
**PE (16:1e) (22:6)**	0.038	0.269	1.49	PI (18:0) (20:4)	0.038	0.269	−1.38
**BiotinylPE (32:5)**	0.004	0.269	1.46	TG (16:0) (16:0) (17:0)	0.038	0.269	−1.39
**PIP2 (31:6e)**	0.002	0.269	1.32	TG (6:0) (6:0) (14:1)	0.038	0.269	−1.44
**DG (8:0) (12:2)**	0.050	0.377	1.27	TG (12:1e) (6:0) (6:0)	0.002	0.269	−1.46
**DG (4:0) (10:3)**	0.011	0.269	1.20	PE (18:0) (22:6)	0.011	0.269	−1.47
**DG (4:0) (11:3)**	0.038	0.269	1.12	CerP (d36:3+O)	0.016	0.338	−1.53
**PC (17:0) (14:1)**	0.038	0.269	1.07	PE (16:1e) (20:4)	0.004	0.269	−1.57
**PC (16:1e) (18:1)**	0.030	0.377	−0.91	PC (32:0e)	0.038	0.269	−1.62
**CL (15:0) (18:1) (15:0) (18:1)**	0.030	0.269	−1.16	PC (32:1e)	0.038	0.269	−1.64
**PE (40:5e)**	0.038	0.269	−1.19	PE (18:0) (20:3)	0.011	0.269	−1.74
**DG (6:0) (11:3)**	0.038	0.269	−1.23	SM (d38:2)	0.038	0.269	−1.80
**ChE (20:4)**	0.038	0.269	−1.31	PC (35:1)	0.038	0.269	−2.09
**(c)**							
**Protein**	***p*-Value**	**BH *p*-Value**	**tFC**	**Protein**	***p*-Value**	**BH *p*-Value**	**tFC**
**ACAN**	0.0126	0.5077	1.79	MICA	0.0113	0.5077	0.90
**CFL1**	0.0407	0.5077	1.62	HAPLN1	0.0424	0.5077	0.86
**CXCL11**	0.0465	0.5077	1.46	PRKCA	0.0351	0.5077	−1.02
**H2AFZ**	0.0191	0.5077	1.44	PTPN6	0.0440	0.5077	−1.04
**MUC1**	0.0370	0.5077	1.39	IGFBP2	0.0285	0.5077	−1.07
**NAMPT**	0.0287	0.5077	1.36	FSTL1	0.0370	0.5077	−1.12
**INS**	0.0185	0.5077	1.35	CTSD	0.0208	0.5077	−1.15
**CD97**	0.0058	0.5077	1.32	PROC	0.0148	0.5077	−1.21
**ICOS**	0.0275	0.5077	1.31	CCL28	0.0086	0.5077	−1.21
**PARK7**	0.0278	0.5077	1.31	CHRDL1	0.0258	0.5077	−1.22
**FAM107B**	0.0279	0.5077	1.29	MSN	0.0142	0.5077	−1.24
**CD38**	0.0478	0.5077	1.29	MMP14	0.0278	0.5077	−1.29
**NGF**	0.0079	0.5077	1.20	CCL2	0.0300	0.5077	−1.34
**PPIF**	0.0152	0.5077	1.17	CXCL6	0.0167	0.5077	−1.36
**THPO**	0.0100	0.5077	1.12	GNLY	0.0453	0.5077	−1.43
**DCN**	0.0127	0.5077	1.09	CTSV	0.0370	0.5077	−1.47

^1^ Metabolites associated with bacteria.

**Table 3:** Baseline Characteristics of HiPOR Delirium Cases and No Delirium Controls.

Characteristics	Proteomics		Metabolomics	
	D (n = 24)	C (n = 24)	D (n = 18)	C (n = 18)
Age, M (SD) (Range)	73.0 (4.9) (65–81)	72.6 (5.5) (64–83)	72.9 (5.1) (65–81)	73.0 (5.7) (65–83)
Female, n (%)	11 (46)	11 (46)	10 (56)	10 (56)
MMSE, M (SD)	27.2 (2.0)	27.5 (1.7)	27.3 (2.1)	27.7 (1.4)
Absolute difference in year of surgery between D and C, mean (SD)	0.8 (0.8)		1.2 (1.2)	
**Characteristics**	**Lipidomics**		**Multi-omics**	
	**D (n= 16)**	**C (n = 16)**	**D (n = 15)**	**C (n = 15)**
Age, M (SD) (Range)	72.9 (4.6) (65–81)	72.6 (5.3) (64–82)	73.0 (4.8) (65–81)	72.3 (5.4) (64–82)
Female, n (%)	9 (56)	9 (56)	8 (53)	8 (53)
MMSE, M (SD)	27.2 (2.2)	27.8 (1.5)	27.4 (2.1)	27.9 (1.3)
Absolute difference in year of surgery between D and C, mean (SD)	1.0 (0.8)		0.9 (0.8)	

Abbreviations: C = control (no delirium), D = delirium, M = mean, SD = standard deviation.

**Table 4:** Summary of counts by molecule type used for downstream analysis and the quality control (QC) strategy deployed. We performed downstream analysis using molecules with significant nominal *p*-values (<0.05).

Molecular Type	All ^1^	Used for Analysis	QC Approach	<10% CV ^2^ n (%)
**Lipids**	161	26	Pooled QC samples [15,34]	159 (98.8)
**Metabolites**	219	51	Pooled QC samples [15,34]	219 (100)
**Proteins**	1305	32	Multi-step QC process established by manufacturer [33]	N/A

^1^ Count after quantitative acquisition; ^2^ Coefficient of Variation.

**Table 5:** Breakdown of the classes for the significantly differentially expressed lipids. The down and up columns represent the number of lipids downregulated and upregulated, respectively, in that class in the delirium group when compared to the control group.

Lipid Class	Total (n = 26)	Down (n = 18)	Up (n = 8)
Phosphatidylcholine	6	4	2
Diacylglycerols	5	2	3
Phosphatidylethanolamines	5	4	1
Triacylglycerol	3	3	0
Biotintl-PE	1	0	1
Ceramide-1-phosphates	1	1	0
Cholesteryl esters	1	1	0
Cardiolipins	1	1	0
Phosphatidylinositol	1	1	0
Phosphatidylinositol bisphosphate	1	0	1
Sphingomyelin	1	1	0

**Table 6:** Pathways that were significantly enriched using MetaboAnalyst.

Pathway	Total	Exp	Hits	Raw *p*-Value	FDR
Pentose phosphate pathway	22	0.64	7	1.28 × 10^−6^	0.00011
Arginine biosynthesis	14	0.41	5	2.65 × 10^−5^	0.00111
Glycolysis/Gluconeogenesis	26	0.75	5	0.00067	0.0188
Alanine, aspartate, and glutamate metabolism	28	0.81	5	0.00096	0.0202
Butanoate metabolism	15	0.43	3	0.00805	0.113
Nicotinate and nicotinamide metabolism	15	0.43	3	0.00805	0.113

Abbreviations: Total: number of metabolites in the pathway. Exp: expected number of input metabolites that would be in the pathway by random chance. Hits: metabolites significantly different between delirium and control groups, i.e., input metabolites, that are in the pathway. FDR: False discovery rate based on the Benjamini–Hochberg method [37].

## Data Availability

Processed data used for this research can be found in the Supplementary Materials. Additional data can be obtained from the corresponding author upon reasonable request.

## Supplementary Materials

The following supporting information can be downloaded at: https://www.mdpi.com/article/10.3390/biom14080924/s1, Figure S1: General study design for lipidomics and metabolomics, Figure S2: Patient Selection for Use in Analysis, Figure S3: Boxplots show the measurement distributions for the top three molecules by type, Figure S4: Original pathways generated through Metaboanalyst, Figure S5: The AUCs for the SVM prediction results, Figure S6: Sample Deviation Plot, Figure S7: Elastic Net Regression ROC for the 25 molecules, Figure S8: Elastic Net Regression ROC for the select 16 molecules, Table S1: Stable mass/charge (m/z) values for targeted metabolomics in both positive and negative ionization modes, Table S2: Kyoto Encyclopedia of Genes and Genomes (KEGG) IDs list for targeted metabolites, Table S3: Percentage of molecules with a coefficient of variation (CV) less than noted in the column header, Table S4: List of input molecules for each stage of systems analysis, Table S5: Functional analysis of lipids at the system level using LIPEA, Table S6: Leave-one-out-cross-validation (L1OXV) accuracy and area under curve (AUC) of the receiver’s operating curve (ROC), Table S7: Metabolites common between plasma and CSF, Table S8: Quality Control Measures Applied to Instrumentation
