# Supplementary material for: Integrated Multi-Omics Analysis of Cerebrospinal Fluid in Postoperative Delirium
Source: Biomolecules. 2024 Jul 30;14(8):924. doi: 10.3390/biom14080924 (PMC11352186; doi:10.3390/biom14080924)
Supplement: Supplementary file 1 [file biomolecules-14-00924-s001.zip › Supplemental Figures.pdf]

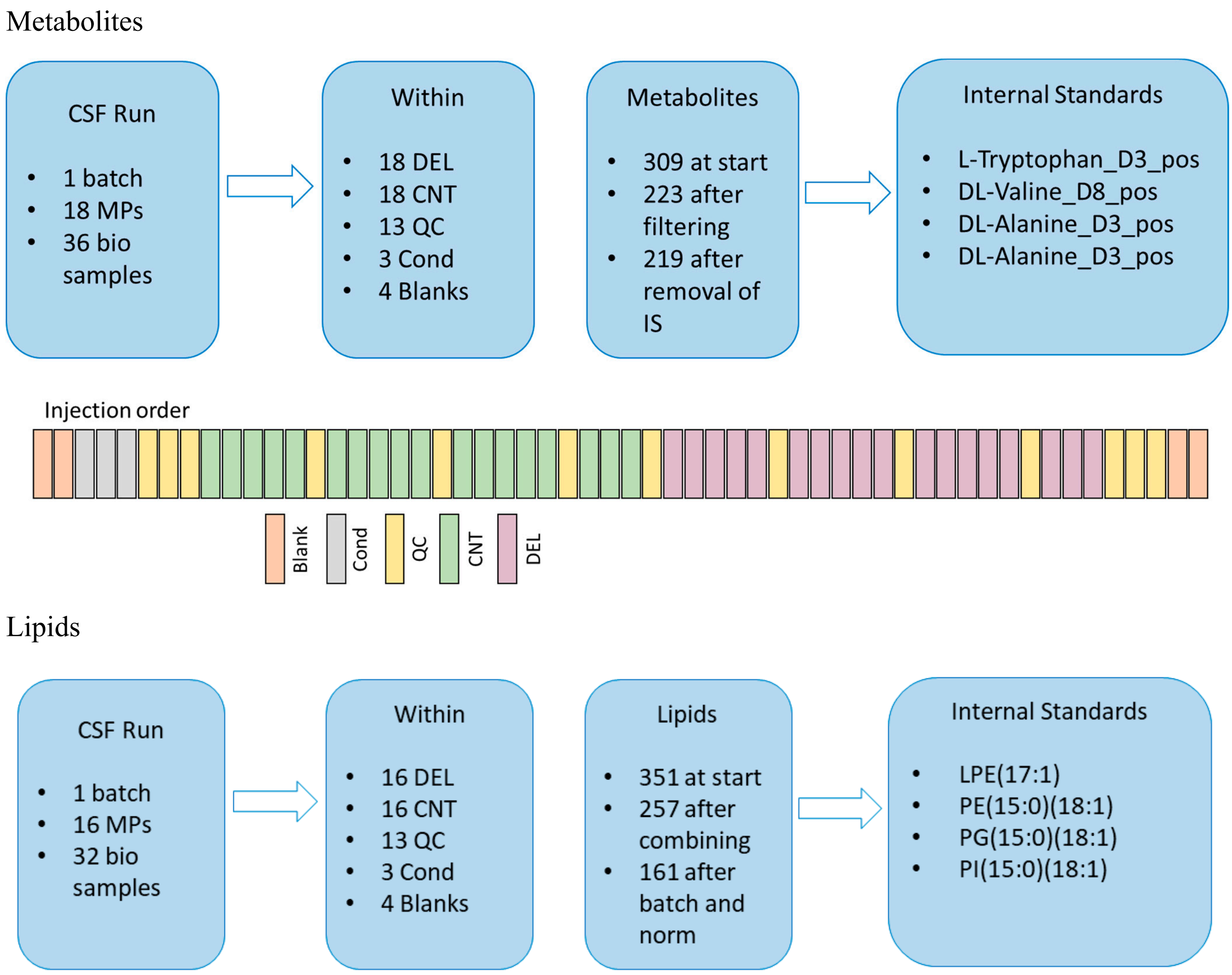

Figure S1: General study design for lipidomics and metabolomics.

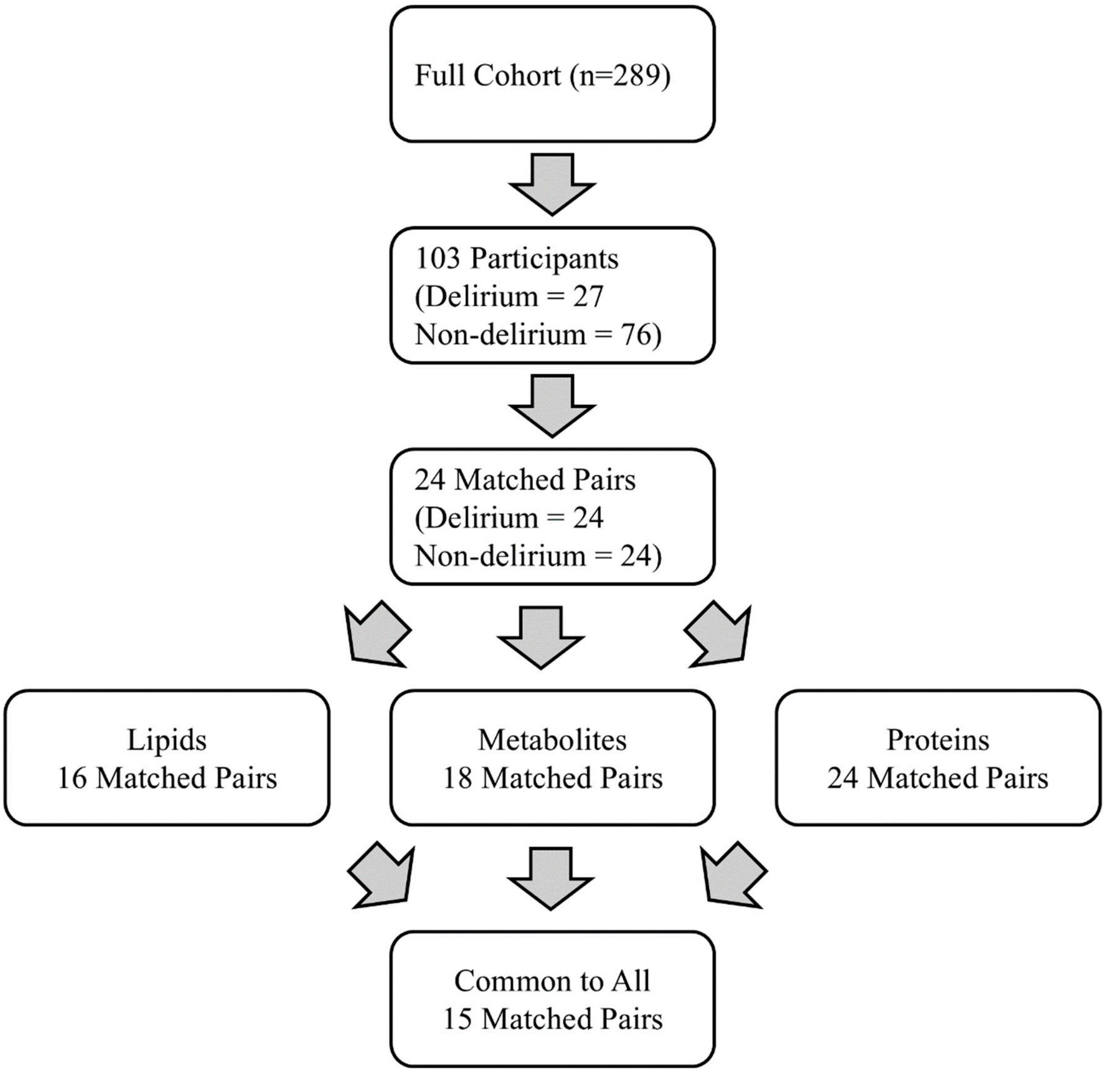

Figure S2: Patient Selection for Use in Analysis. The full cohort of 289 subjects was reduced to 103 prior to the match due to the exclusion of those (i) enrolled before 2009 (to limit sample degradation), (ii) without baseline cognition (MMSE), (iii) with low CSF volume (200 ul), and (iv) with subsyndromal delirium, missing data, or inability to determine delirium status. Applying our matching algorithm with the four matching factors (age within five years, exact sex, year of surgery within two years, MMSE score within three points) to the 103 subjects yielded 24 matched pairs, which were used for proteomic profiling. Due to limited sample volumes and quality limitations, 18 and 16 delirium cases matched with no-delirium controls were processed for metabolomics and lipidomics, respectively. Fifteen matched pairs had all three omics signals measured.

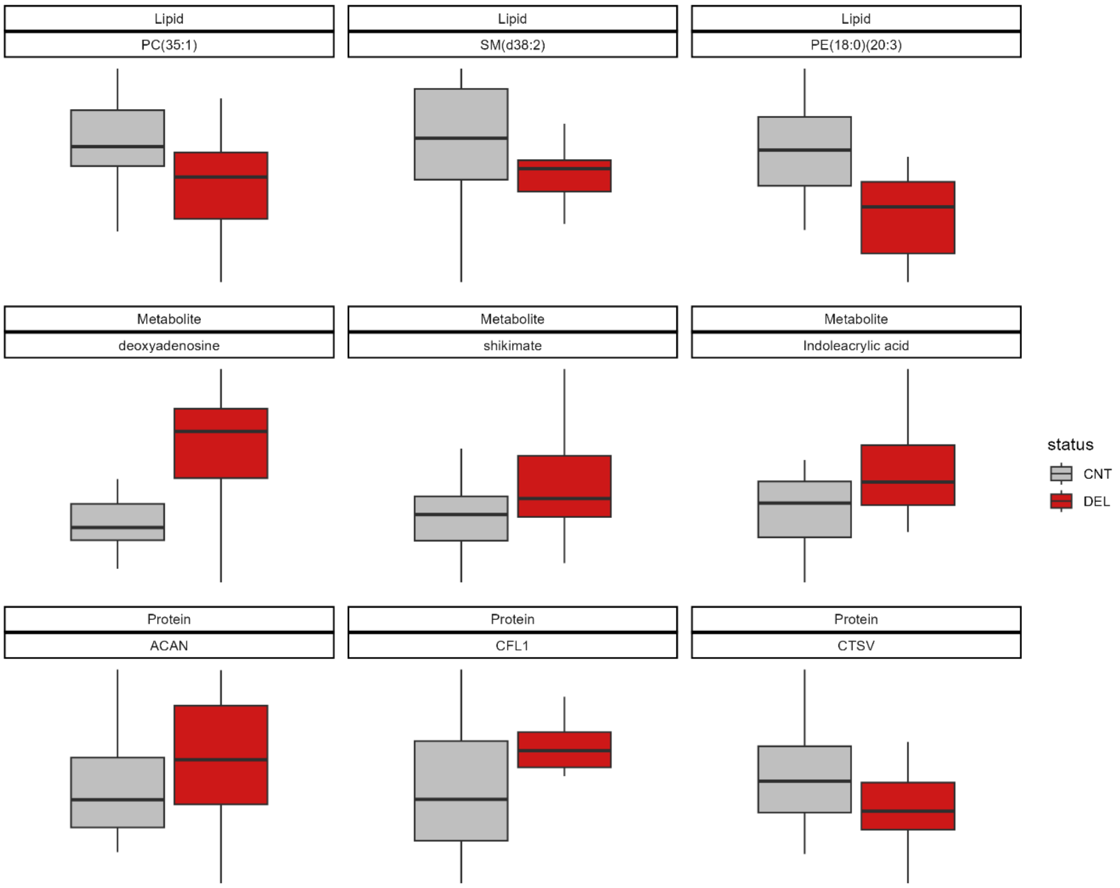

Figure S3: Boxplots show the measurement distributions for the top three molecules by type (top: lipids, middle: metabolites, bottom: proteins) with the highest magnitude fold change (FC) observed between delirium and non-delirium controls. (PC(35:1) FC=-2.09, SM(d38:2) FC=-1.80, PE(18:0)(20:3) FC=-1.74, deoxyadenosine FC=2.46, shikimate FC=2.25, indoleacrylic acid FC = 2.02, ACAN FC=1.79, CFL1 FC=1.62, CTSV FC=-1.47)

a. Arginine synthesis

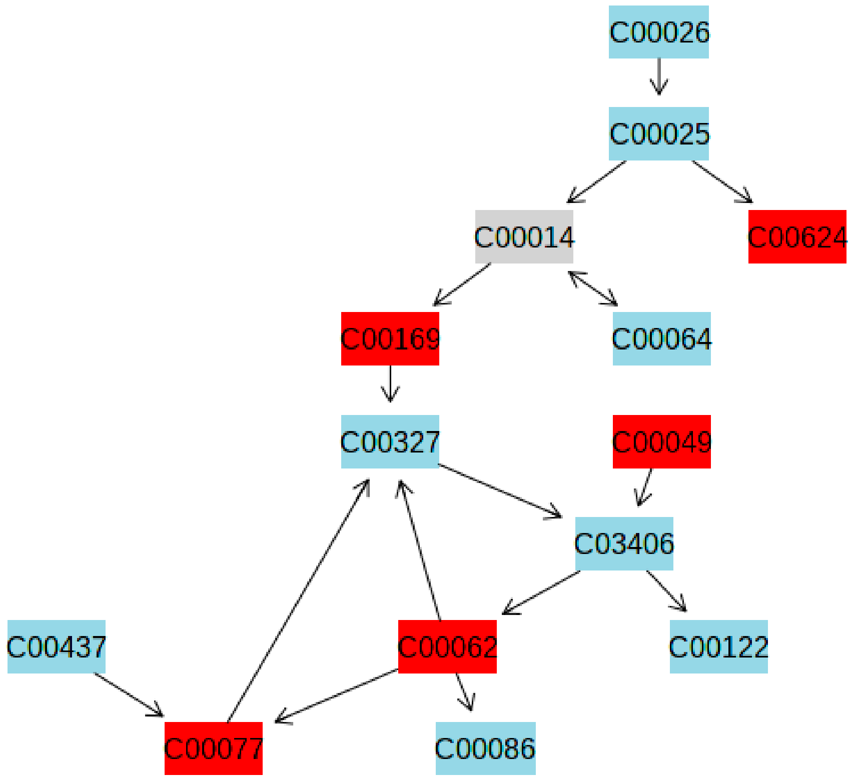

b. Pentose Phosphate Pathway

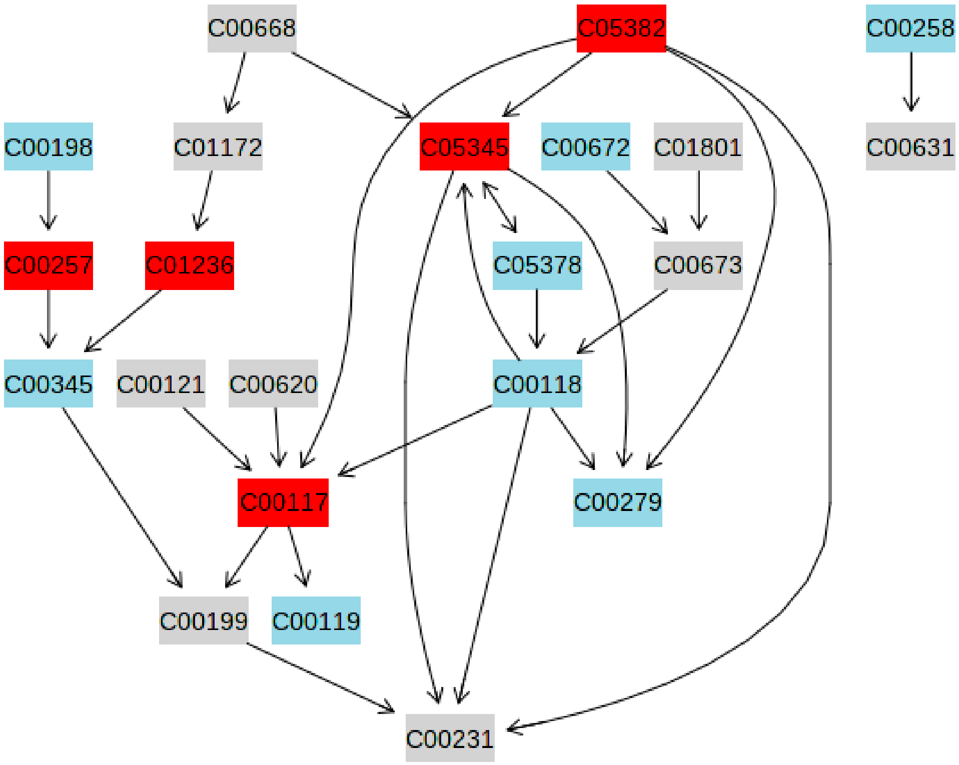

Figure S4: Original pathways generated through Metaboanalyst. These figures were used to generate publication-ready figures that included analyte names.

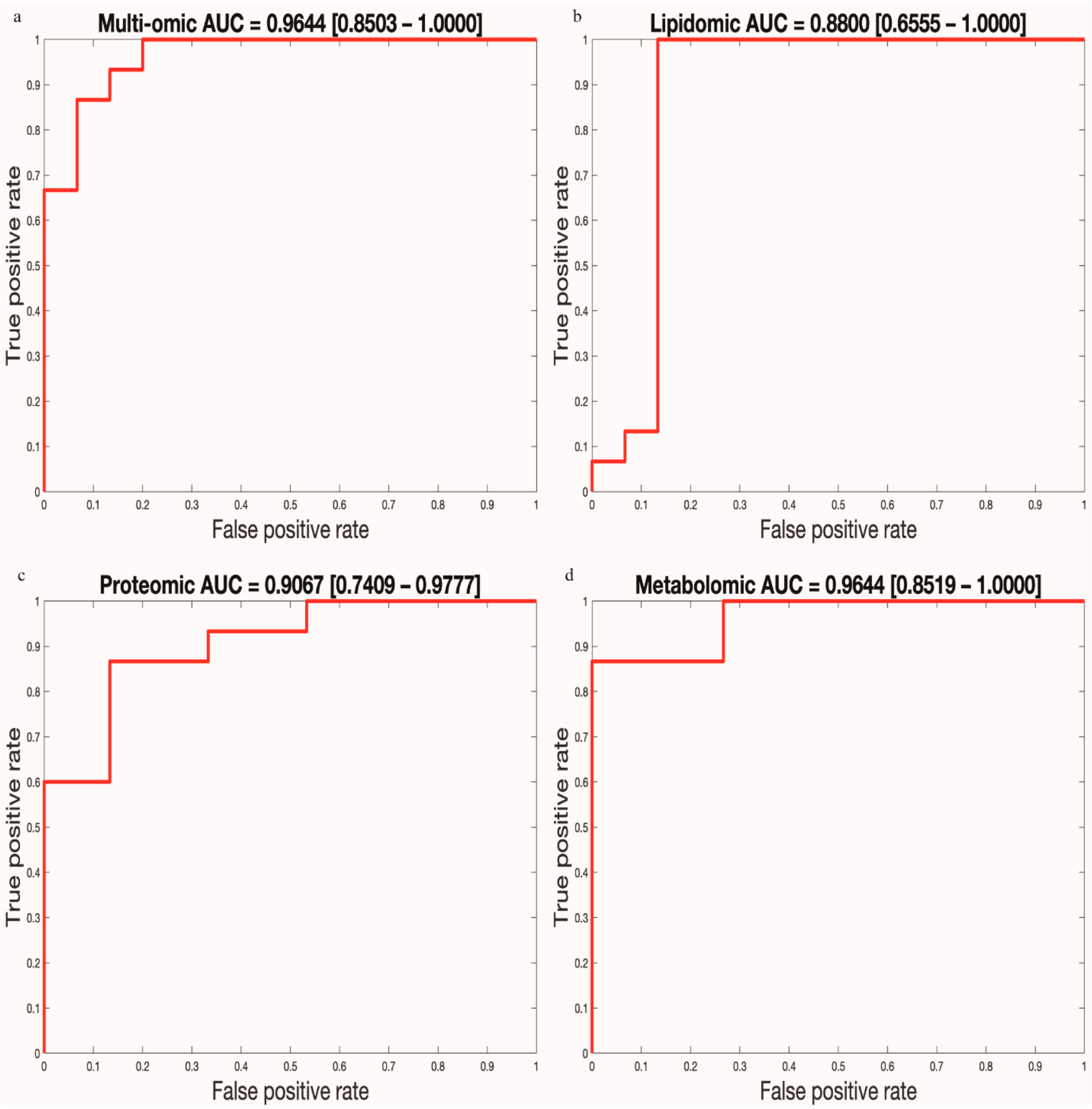

Figure S5: The AUCs for the SVM prediction results for a) multi-omics, b) lipidomics, c) proteomics, and d) metabolomics signatures.

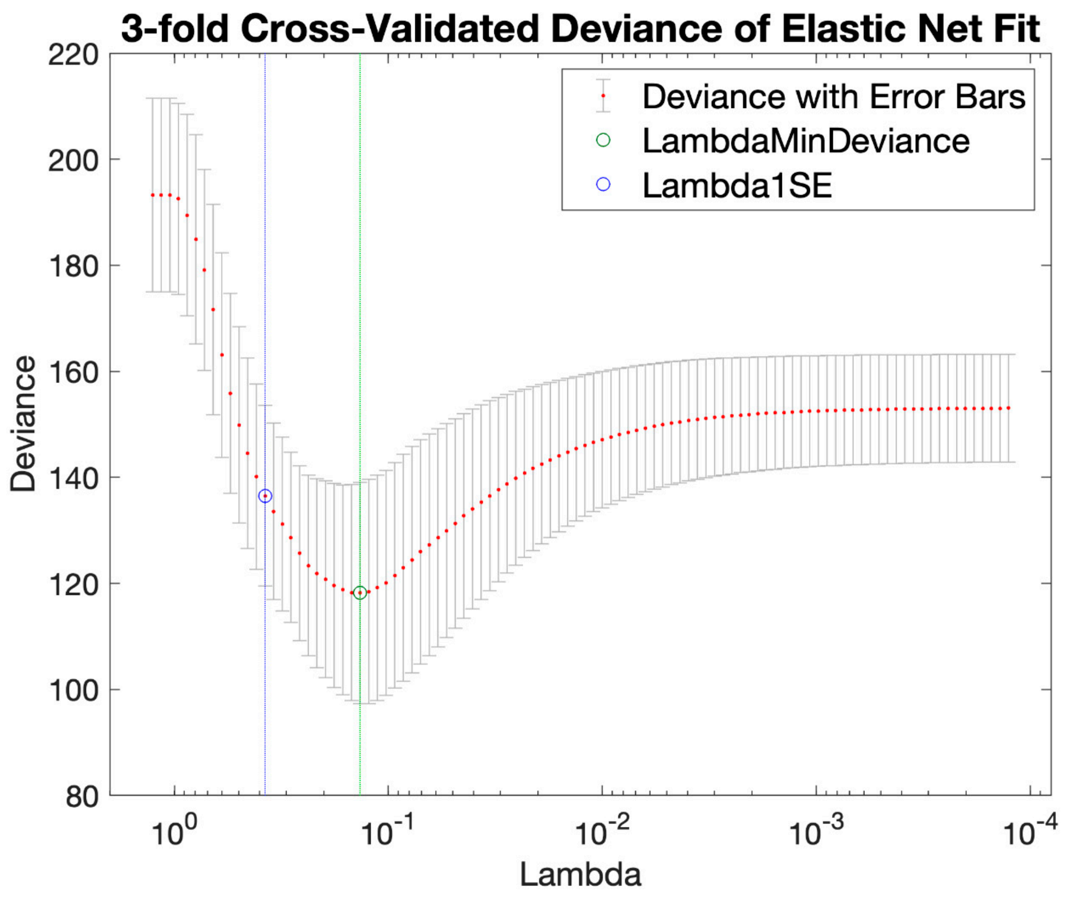

Figure S6: Sample Deviation Plot. Sample Deviance vs. Lambda plot for regularized logistic regression with elastic net analysis. 3-fold cross-validation was used to calculate the deviance (error term) with respect to Lambda (regularization coefficient). The Lambda value that resulted in the minimum deviance was used in the final model that incorporated all the samples.

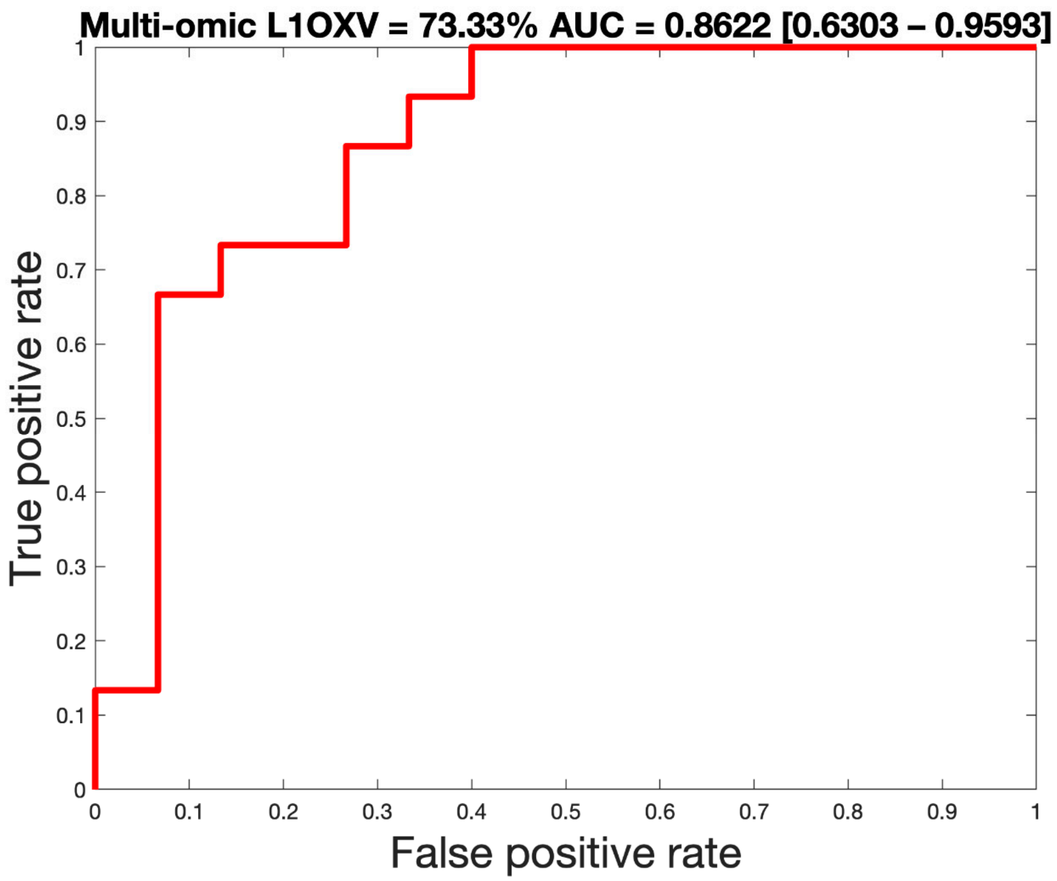

Figure S7: Elastic Net Regression ROC for the 25 molecules.

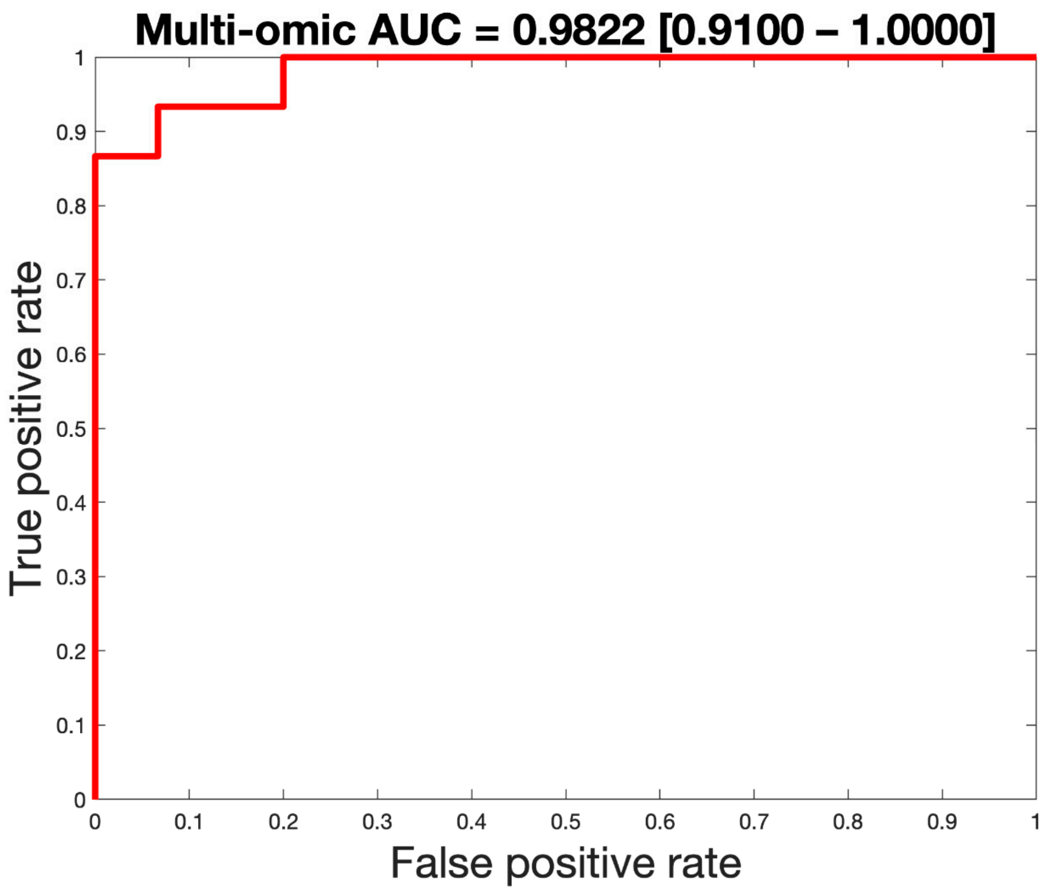

Figure S8: Elastic Net Regression ROC for the select 16 molecules.
