# Supplementary material for: Integrated Multi-Omics Analysis of Cerebrospinal Fluid in Postoperative Delirium
Source: Biomolecules. 2024 Jul 30;14(8):924. doi: 10.3390/biom14080924 (PMC11352186; doi:10.3390/biom14080924)
Supplement: Supplementary file 1 [file biomolecules-14-00924-s001.zip › Supplemental Tables.pdf]

Table S1: Stable mass/charge (m/z) values for targeted metabolomics in both positive and negative ionization modes. The list of targeted molecules and their corresponding mass/charge (m/z) for the positive and negative ion modes (Supplementary Table S1) are used to link to the molecular formula and PubChem Identifier (Supplementary Table S2).

Negative ion mode		Positive ion mode	
**m/z [M-1]-**	**Name**	**m/z [M+1]+**	**Name**
73	glyoxylate	61.1	Urea
75	glycolate	62.1	ethanolamine
87	pyruvate	76.1	glycine
89	lactate	89	putrescine
101	2-oxobutanoate	90.04	sarcosine
101.05	acetoacetate	90.1	alanine
103.04	3-hydroxybuterate	102	betaine aldehyde
105	glycerate	104	choline
111.05	uracil	104.01	4-aminobutyrate
115	fumarate	104.02	dimethylglycine
115.03	Maleic acid	106	serine
115.05	2-keto-isovalerate	112.1	cytosine
116.001	Guanidoacetic acid	114	Creatinine
117	succinate	116.1	proline
117.002	Methylmalonic acid	118	indole
119.02	3-S-methylthiopropionate	118.02	betaine
122	nicotinate	118.1	valine
124	taurine	120	threonine
128	Pyroglutamic acid	120.15	homoserine
129.003	Citraconic acid	121	purine
129.005	2-ketohaxanoic acid	122.1	cysteine
129.1	itaconic acid	123.1	nicotinamide
130	N-Acetyl-L-alanine	127.002	Imidazoleacetic acid
131	oxaloacetate	127.1	thymine
131	Gluterate	130	DL-Pipecolic acid
131.006	Hydroxyisocaproic acid	131.001	N-Acetylputrescine
133	malate	131.9	N-acetyl-L-alanine
135	hypoxanthine	132.003	creatine
136	anthranilate	132.004	hydroxyproline
136.05	p-aminobenzoate	132.1	leucine-isoleucine
137	p-hydroxybenzoate	133	ornithine
139	acetylphosphate	133.1	asparagine
140	Carbamoyl phosphate	134	aspartate
145	a-ketoglutarate	136	adenine
145.004	Phenylpropiolic acid	136.02	Methylcysteine
147	2-oxo-4-methylthiobutanoate	136.12	homocysteine

147.001	2-Hydroxy-2-methylbutanedioic acid	137.001	methylnicotinamide
147.1	2-hydroxyglutarate	142.1	histidinol
149.002	3-methylphenylacetic acid	146	spermidine
151	xanthine	147	lysine
151.004	Hydroxyphenylacetic acid	147.1	glutamine
153	2,3-dihydroxybenzoic acid	148	O-acetyl-L-serine
155	orotate	148.1	glutamate
157	dihydroorotate	149.35	Mevalonate
157.05	allantoin	150.1	methionine
159	2-oxoadipate	152.2	guanine
160.001	Aminoadipic acid	154.05	cysteine sulfinate
160.002	Indole-3-carboxylic acid	156.1	histidine
163	phenylpyruvate	160	2-Aminooctanoic acid
165.004	Atrolactic acid	162.1	carnitine
165.006	Phenyllactic acid	166	Methionine sulfoxide
166	quinolinate	166.1	phenylalanine
167	phosphoenolpyruvate	169	Pyridoxamine
167.001	Uric acid	170	pyridoxine
169	dihydroxy-acetone-phosphate	170.1	1-Methyl-Histidine
169.05	D-glyceraldehdye-3-phosphate	175	N-acetyl-L-ornithine
171	sn-glycerol-3-phosphate	175.02	arginine
171.1	glycerol 3-phosphate	176	citrulline
173	shikimate	176	N-acetyl-L-aspartic acid
173	dehydroascorbic acid	177.05	N-carbamoyl-L-aspartate
173.05	aconitate	180	glucosamine
175	allantoate	182.1	tyrosine
175.001	Ascorbic acid	184.001	Phosphorylcholine
175.002	2-Isopropylmalic acid	186	3-phospho-serine
175.03	N-carbamoyl-L-aspartate-nega	188	N-acetyl spermidine
176.8	Pyrophosphate	189.001	N6-Acetyl-L-lysine
177	glucono-delta-lactone	189.002	Acetyllysine
178.07	hippurate	189.1	N-acetyl-glutamine
179	myo-inositol	190.1	N-acetyl-glutamate
179.05	hydroxyphenylpyruvate	203	Ng,NG-dimethyl-L-arginine
181	sorbitol	203.1	spermine
182	homocysteic acid	204	Acetylcarnitine DL
182.003	4-Pyridoxic acid	205	tryptophan
185	3-phosphoglycerate	209	Kynurenine
186	Indoleacrylic acid	212	phosphocreatine

188	Kynurenic acid	222	N-acetyl-glucosamine
191	citrate-isocitrate	223	Flavone
191.02	isocitrate	223	cystathionine
191.05	citrate	235	5-methoxytryptophan
193	2-dehydro-D-gluconate	241.002	Cystine
195	D-gluconate	244.1	cytidine
199	D-erythrose-4-phosphate	245	N-acetyl spermine
204.001	Xanthurenic acid	245.1	biotin
205	lipoate	252	deoxyadenosine
209	D-glucarate	255	Nicotinamide Riboside
213	deoxyribose-phosphate	258.1	Glycerophosphocholine
218	pantothenate	259	acadesine
225	prephenate	260	D-glucosamine-6-phosphate
227	deoxyuridine	260.1	D-glucosamine-1-phosphate
229	ribose-phosphate	264	AL-II
241	thymidine	265	thiamine
243	uridine	268	S-ribosyl-L-homocysteine-posi
243.2	2-deoxyglucose-6-phosphate	268.1	deoxyguanosine
251	deoxyinosine	268.15	adenosine
253.1	shikimate-3-phosphate	281.8	1-Methyladenosine
257	D-glucono-?-lactone-6-phosphate	284.1	guanosine
259	hexose-phosphate	291	L-arginino-succinate
259.01	glucose-1-phosphate	291.2	arginosuccinic acid
259.02	glucose-6-phosphate	294	AL-I
259.03	fructose-6-phosphate	298	S-methyl-5-thioadenosine
265	1,3-diphopshateglycerate	298.002	7-methylguanosine
265.1	2,3-Diphosphoglyceric acid	301.1	retinoic acid
266	S-ribosyl-L-homocysteine-nega	305	N-acetylaspartylglutamic acid
267	inosine	308	dCMP
275	6-phospho-D-gluconate	308.1	glutathione
283	xanthosine	323	dTMP
289	D-sedoheptulose-1-7-phosphate	324	CMP
298.07	4-phosphopantothenate	325	UMP
300	N-acetyl-glucosamine-1-phosphate	332.1	dAMP
306	glutathione-nega	335	Nicotinamide ribotide

307	dUMP-nega	339	aminoimidazole carboxamide ribonucleotide
313	Geranyl-PP	345.2	thiamine-phosphate
319	O8P-O1P	348.1	dGMP
321	dTMP-nega	348.15	AMP
328	cyclic-AMP	349	IMP
339	fructose-1,6-bisphosphate	355	S-adenosyl-L-methioninamine
341	trehalose-sucrose	364	GMP
341.1	Cellobiose	365	xanthosine-5-phosphate
367	orotidine-5-phosphate	377	riboflavin
369	SBP	385.1	S-adenosyl-L-homoCysteine-posi
381	trans, trans-farnesyl diphosphate	387.36	cholesterol
383.1	S-adenosyl-L-homocysteine-nega	399.1	S-adenosyl-L-methionine
386	dCDP-nega	442	folate
389	5-phosphoribosyl-1-pyrophosphate	444.2	7,8-dihydrofolate
391.202	Deoxycholic acid	460.1	5-methyl-THF
399	OBP	464	Adenylosuccinate
401	dTDP-nega	525.5	Diiodothyronine
402	CDP-nega	613	glutathione disulfide-posi
403	UDP-nega	664.1	NAD+_posi
407.2	Cholic acid	666.1	NADH
421	trehalose-6-Phosphate	688	dephospho-CoA-posi
423.1	Thiamine pyrophosphate	744.2	NADP+_posi
426	adenosine 5-phosphosulfate	746.15	NADPH
426.1	ADP-nega	768	coenzyme A-posi
426.12	dGDP-nega	786	FAD
427	IDP-nega	810	acetyl-CoA-posi
442	GDP-nega	824.1	propionyl-CoA-posi
445	CDP-ethanolamine	852	acetoacetyl-CoA-posi
455	FMN	854	malonyl-CoA-posi
465.2	cholesteryl sulfate	868.1	succinyl-CoA-posi
466	dCTP-nega	912.17	HMG-CoA_pos
467	dUTP-nega		
481	dTTP-nega		
482	CTP-nega		
483	UTP-nega		
487	CDP-choline		
490	dATP-nega		

498.2	Taurodeoxycholic acid		
506.1	ATP-nega		
506.12	dGTP		
522	GTP-nega		
535.4	UDP-xylose		
565	UDP-D-glucose		
579	UDP-D-glucuronate		
588	ADP-D-glucose		
602	guanosine 5-diphosphate,3-diphosphate		
606	UDP-N-acetyl-glucosamine		
611	glutathione disulfide-nega		
662	NAD+_nega		
664	NADH-nega		
686.2	dephospho-CoA-nega		
689	cyclic bis(3->5) dimeric GMP		
742	NADP+_nega		
744	NADPH-nega		

Table S2: Kyoto Encyclopedia of Genes and Genomes (KEGG) IDs list for targeted metabolites. The list of targeted molecules and their corresponding mass/charge (m/z) are used to link to the molecular formula and PubChem Identifier.

Metabolite name	formula	PubChem Identifier (KEGG/HMDB)
1,3-diphopshateglycerate	C3H8O10P2	C00236
1-Methyladenosine	C11H15N5O4	C02494
1-Methyl-Histidine	C7H11N3O2	C01152
2,3-dihydroxybenzoic acid	C7H6O4	C00196
2,3-Diphosphoglyceric acid	C3H8O10P2	C01159
2-Aminooctanoic acid	C8H17NO2	HMDB00991
2-dehydro-D-gluconate	C6H10O7	C00629
2-deoxyglucose-6-phosphate	C6H13O8P	C06369
2-Hydroxy-2-methylbutanedioic acid	C5H8O5	C02612
2-hydroxygluterate	C5H8O5	C02630
2-Isopropylmalic acid	C7H12O5	C02504
2-ketohaxanoic acid	C6H10O3	HMDB01864
2-keto-isovalerate	C5H8O3	C00141
2-oxo-4-methylthiobutanoate	C5H8O3S	C01180
2-oxoadipate	C6H8O5	C00322
2-oxobutanoate	C4H6O3	C00109
3-hydroxy-3-methylglutaryl-CoA	C27H44N7O20P3S	C00356
3-hydroxybuteric acid	C4H8O3	C01089
3-hydroxybutyryl-CoA	C25H42N7O18P3S	C01144
3-methylphenylacetic acid	C9H10O2	HMDB02222
3-phosphoglycerate	C3H7O7P	C00197
3-phosphoserine	C3H8NO6P	C01005
3-S-methylthiopropionate	C4H8O2S	C08276
4-aminobutyrate	C4H9NO2	C00334
4-phosphopantothenate	C9H18NO8P	C03492
4-Pyridoxic acid	C8H9NO4	C00847
5-methoxytryptophan	C12H14N2O3	HMDB02339
5-methyl-THF	C20H25N7O6	C00440
5-phosphoribosyl-1-pyrophosphate	C5H13O14P3	C00119
6-phospho-D-gluconate	C6H13O10P	C00345
7,8-dihydrofolate	C19H21N7O6	C00415
7-methylguanosine	C11H16N5O5	HMDB01107
acadesine	C9H14N4O5	D02742
acetoacetate	C4H6O3	C00164
acetoacetyl-CoA	C25H40N7O18P3S	C00332
Acetylcarnitine DL	C9H18NO4	C02571
acetyl-CoA	C23H38N7O17P3S	C00024
Acetyllysine	C8H16N2O3	C02727
acetylphosphate	C2H5O5P	C00227
aconitate	C6H6O6	C00417
adenine	C5H5N5	C00147
adenosine	C10H13N5O4	C00212

adenosine 5-phosphosulfate	C10H14N5O10PS	C00224
ADP-D-glucose	C16H25N5O15P2	C00498
ADP-nega	C10H15N5O10P2	C00008
a-ketoglutarate	C5H6O5	C00026
alanine	C3H7NO2	C00041
allantoate	C4H8N4O4	C00499
allantoin	C4H6N4O3	C01551
Aminoadipic acid	C6H11NO4	C00956
aminoimidazole carboxamide ribonucleotide	C9H15N4O8P	C04677
AMP	C10H14N5O7P	C00020
anthranilate	C7H7NO2	C00108
arginine	C6H14N4O2	C00062
Ascorbic acid	C6H8O6	C00072
asparagine	C4H8N2O3	C00152
aspartate	C4H7NO4	C00049
ATP-nega	C10H16N5O13P3	C00002
Atrolactic acid	C9H10O3	HMDB00475
betaine	C5H11NO2	C00719
betaine aldehyde	C5H12NO	C00576
biotin	C10H16N2O3S	C00120
butyryl-CoA	C25H42N7O17P3S	C00630
Carbamoyl phosphate	CH4NO5P	C00169
carnitine	C7H15NO3	C00318
CDP-choline	C14H27N4O11P2	C00307
CDP-ethanolamine	C11H20N4O11P2	C00570
CDP-nega	C9H15N3O11P2	C00112
Cellobiose	C12H22O11	C00185
cholesterol	C27H46O	C00187
cholesteryl sulfate	C27H46O4S	HMDB00653
Cholic acid	C24H40O5	C00695
choline	C5H14NO	C00114
Citraconic acid	C5H6O4	C02226
citrate	C6H8O7	C00158
citrate-isocitrate	C6H8O7	C00158
citrulline	C6H13N3O3	C00327
CMP	C9H14N3O8P	C00055
coenzyme A	C21H36N7O16P3S	C00010
creatine	C4H9N3O2	C00300
Creatinine	C4H7N3O	C00791
CTP-nega	C9H16N3O14P3	C00063
cyclic bis(3->5) dimeric GMP	C20H24N10O14P2	C16463
cyclic-AMP	C10H12N5O6P	C00575
cystathionine	C7H14N2O4S	C02291
cysteine	C6H12N2O4S2	C00491
cysteine sulfinate	C3H7NO4S	C00606
Cystine	C6H12N2O4S2	C00491
cytidine	C9H13N3O5	C00475

cytosine	C4H5N3O	C00380
dAMP	C10H14N5O6P	C00360
dATP-nega	C10H16N5O12P3	C00131
dCDP-nega	C9H15N3O10P2	C00705
dCMP	C9H14N3O7P	C00239
dCTP-nega	C9H16N3O13P3	C00458
deoxyadenosine	C10H13N5O3	C00559
Deoxycholic acid	C26H43NO5	C04483
deoxyguanosine	C10H13N5O4	C00330
deoxyinosine	C10H12N4O4	C05512
deoxyribose-phosphate	C5H11O7P	C00673
deoxyuridine	C9H12N2O5	C00526
dephospho-CoA	C21H35N7O13P2S	C00882
D-erythrose-4-phosphate	C4H9O7P	C00279
dGDP-nega	C10H15N5O10P2	C00361
D-glucarate	C6H10O8	C00818
D-gluconate	C6H12O7	C00257
D-glucono-?-lactone-6-phosphate	C6H11O9P	C01236
D-glucosamine-1-phosphate	C6H14NO8P	C00352
D-glucosamine-6-phosphate	C6H14NO8P	C00352
D-glyceraldehdye-3-phosphate	C3H7O6P	C00118
dGMP	C10H14N5O7P	C00362
dGTP	C10H16N5O13P3	C00286
dihydroorotate	C5H6N2O4	C00337
dihydroxy-acetone-phosphate	C3H7O6P	C00111
Diiodothyronine	C15H13I2NO4	HMDB00582
dimethylglycine	C4H9NO2	C01026
DL-Pipecolic acid	C6H11NO2	C00408
D-sedoheptulose-1-7-phosphate	C7H15O10P	C05382
dTDP	C10H16N2O11P2	C00363
dTMP	C10H15N2O8P	C00364
dTTP	C10H17N2O14P3	C00459
dUMP	C9H13N2O8P	C00365
dUTP	C9H15N2O14P3	C00460
ethanolamine	C2H7NO	C00189
FAD	C27H33N9O15P2	C00016
Flavone	C15H10O2	C15608
FMN	C17H21N4O9P	C00061
folate	C19H19N7O6	C00504
fructose-1,6-bisphosphate	C6H14O12P2	C05378
fructose-6-phosphate	C6H13O9P	C05345
fumarate	C4H4O4	C00122
GDP-nega	C10H15N5O11P2	C00035
Geranyl-PP	C10H20O7P2	C00341
glucono-?-lactone	C6H10O6	C00198
glucosamine	C6H13NO5	C00329
glucose-1-phosphate	C6H13O9P	C00103

glucose-6-phosphate	C6H13O9P	C00668
glutamate	C5H9NO4	C00025
glutamine	C5H10N2O3	C00064
glutarate	C5H8O4	C00489
glutathione	C10H17N3O6S	C00051
glutathione disulfide	C20H32N6O12S2	C00127
glycerate	C3H6O4	C00258
glycerol 3-phosphate	C3H9O6P	C00093
Glycerophosphocholine	C8H21NO6P	C00670
glycine	C2H5NO2	C00037
glycolate	C2H4O3	C00160
glyoxylate	C2H2O3	C00048
GMP	C10H14N5O8P	C00144
GTP	C10H16N5O13P3	C00044
Guanidoacetic acid	C3H7N3O2	C00581
guanine	C5H5N5O	C00242
guanosine	C10H13N5O5	C00387
guanosine 5-diphosphate,3-diphosphate	C10H11N5O17P4	C01228
hexose-phosphate	C6H13O9P	C05345
histidine	C6H9N3O2	C00135
histidinol	C6H11N3O	C00860
HMG-CoA	C27H44N7O20P3S	C00356
homocysteic acid	C4H9NO5S	C16511
homocysteine	C4H9NO2S	C00155
homoserine	C4H9NO3	C00263
Hydroxyisocaproic acid	C6H12O3	HMDB00746
Hydroxyphenylacetic acid	C8H8O3	C05852
hydroxyphenylpyruvate	C9H8O4	C01179
hydroxyproline	C5H9NO3	C01157
hypoxanthine	C5H4N4O	C00262
IDP-nega	C10H14N4O11P2	C00104
Imidazole	C3H4N2	C01589
Imidazoleacetic acid	C6H8N2O2	C02835
IMP	C10H13N4O8P	C00130
indole	C8H7N	C00463
Indole-3-carboxylic acid	C9H7NO2	HMDB03320
Indoleacrylic acid	C11H9NO2	HMDB00734
inosine	C10H12N4O5	C00294
isocitrate	C6H8O7	C00311
itaconic acid	C5H6O4	HMDB0002092
Kynurenic acid	C10H7NO3	C01717
Kynurenine	C10H12N2O3	C00328
lactate	C3H6O3	C00186
L-arginino-succinate	C10H18N4O6	C03406
leucine-isoleucine	C6H13NO2	C00123
lipoate	C8H14O2S2	C00725
lysine	C6H14N2O2	C00047

malate	C4H6O5	C00149
Maleic acid	C4H4O4	C01384
malonyl-CoA	C24H38N7O19P3S	C00083
methionine	C5H11NO2S	C00073
Methionine sulfoxide	C5H11NO3S	HMDB02005
Methylcysteine	C4H9NO2S	HMDB0002108
Methylmalonic acid	C4H6O4	C02170
methylnicotinamide	C7H9N2O	C02918
Mevalonate	C6H12O4	C00418
myo-inositol	C6H12O6	C00137
N6-Acetyl-L-lysine	C8H16N2O3	C02727
N-acetyl spermidine	C9H21N3O	C00612
N-acetyl spermine	C12H28N4O	C02567
N-acetyl-aspartylglutamic acid	C11H16N2O8	C12270
N-acetyl-glucosamine	C8H15NO6	C00140
N-acetyl-glucosamine-1-phosphate	C6H14NO8P	C04256
N-acetyl-glutamate	C7H11NO5	C00624
N-acetyl-glutamine	C7H12N2O4	HMDB06029
N-Acetyl-L-alanine	C5H9NO3	C01073
N-acetyl-L-aspartic acid	C6H9NO5	C01042
N-acetyl-L-ornithine	C7H14N2O3	C00437
N-Acetylputrescine	C6H14N2O	C02714
NAD+	C21H27N7O14P2	C00003
NADH	C21H29N7O14P2	C00004
NADP+	C21H28N7O17P3	C00006
NADPH	C21H30N7O17P3	C00005
N-carbamoyl-L-aspartate	C5H8N2O5	C00438
Ng,NG-dimethyl-L-arginine	C8H18N4O2	C03626
nicotinamide	C6H6N2O	C00153
nicotinamide riboside	C11H15N2O5	C03150
Nicotinamide ribotide	C11H15N2O8P	C00455
nicotinate	C6H5NO2	C00253
O8P-O1P (octulose-8-phosphate)	C5H11O8P	
O-acetyl-L-serine	C5H9NO4	C00979
octulose-1,8-bisphosphate (OBP)	C5H11O8P	
ornithine	C5H12N2O2	C00077
orotate	C5H4N2O4	C00295
orotidine-5-phosphate	C10H13N2O11P	C01103
oxaloacetate	C4H4O5	C00036
p-aminobenzoate	C7H7NO2	C00568
pantothenate	C9H17NO5	C00864
phenylalanine	C9H11NO2	C00079
Phenyllactic acid	C9H10O3	C01479
Phenylpropiolic acid	C9H6O2	HMDB00563
phenylpyruvate	C9H8O3	C00166
Phosphocreatine	C4H10N3O5P	C02305
phosphoenolpyruvate	C3H5O6P	C00074

Phosphorylcholine	C5H15NO4P	C00588
p-hydroxybenzoate	C7H6O3	C00156
prephenate	C10H10O6	C00254
proline	C5H9NO2	C00148
propionyl-CoA	C24H40N7O17P3S	C00100
purine	C5H4N4	C00465
putrescine	C4H12N2	C00134
Pyridoxamine	C8H12N2O2	C00534
pyridoxine	C8H11NO3	C00314
Pyroglutamic acid	C5H7NO3	C01879
Pyrophosphate	P2H4O7	C00013
pyruvate	C3H4O3	C00022
quinolinate	C7H5NO4	C03722
retinoic acid	C20H28O2	C00777
riboflavin	C17H20N4O6	C00255
ribose-phosphate	C5H11O8P	C00117
S-adenosyl-L-homocysteine	C14H20N6O5S	C00021
S-adenosyl-L-methioninamine	C14H23N6O3S	C01137
S-adenosyl-L-methionine	C15H22N6O5S	C00019
sarcosine	C3H7NO2	C00213
sedoheptulose 1,7-bisphosphate (SBP)	C7H16O13P2	C00447
serine	C3H7NO3	C00065
shikimate	C7H10O5	C00493
shikimate-3-phosphate	C7H11O8P	C03175
S-methyl-5-thioadenosine	C11H15N5O3S	C00170
sn-glycerol-3-phosphate	C3H9O6P	C00093
sorbitol	C6H14O6	C00794
spermidine	C7H19N3	C00315
spermine	C10H26N4	C00750
S-ribosyl-L-homocysteine	C9H17NO6S	C03539
succinate	C4H6O4	C00042
succinyl-CoA-methylmalonyl-CoA	C25H40N7O19P3S	C00091
succinyl-CoA-posi	C25H40N7O19P3S	C00091
taurine	C2H7NO3S	C00245
Taurodeoxycholic acid	C26H45NO6S	C05463
thiamine	C12H16N4OS	C00378
Thiamine pyrophosphate	C12H19N4O7P2S	C00068
thiamine-phosphate	C12H17N4O4PS	C01081
threonine	C4H9NO3	C00188
thymidine	C10H14N2O5	C00214
thymine	C5H6N2O2	C00178
trans, trans-farnesyl diphosphate	C15H28O7P2	C00448
trehalose-6-Phosphate	C12H23O14P	C00689
trehalose-sucrose	C12H22O11	C00089
tryptophan	C11H12N2O2	C00078
tyrosine	C9H11NO3	C00082
UDP-D-glucose	C15H24N2O17P2	C00029

UDP-D-glucuronate	C15H22N2O18P2	C00167
UDP-N-acetyl-glucosamine	C17H27N3O17P2	C00043
UDP-nega	C9H14N2O12P2	C00015
UDP-xylose	C14H22N2O16P2	C00190
UMP	C9H13N2O9P	C00105
uracil	C4H4N2O2	C00106
Urea	CH4N2O	C00086
Uric acid	C5H4N4O3	C00366
uridine	C9H12N2O6	C00299
UTP-nega	C9H15N2O15P3	C00075
valine	C5H11NO2	C00183
xanthine	C5H4N4O2	C00385
xanthosine	C10H12N4O6	C01762
xanthosine-5-phosphate	C10H13N4O9P	C00655
Xanthurenic acid	C10H7NO4	C02470

Table S3: Percentage of molecules with a coefficient of variation (CV) less than noted in the column header. Corrected represents drift-corrected signal values, and 'processed' represents final signal values used for downstream statistical analysis after preprocessing.

Lipids

CV%	<5	<10	<15	<20	<25	<30	<35	<40	<50	<60	<70	<80	<90	<100
Raw	0.00	0.43	4.72	9.01	13.7	21.5	34.8	44.6	56.7	62.7	69.1	73.4	79.4	83.3
Corrected	0.43	6.87	12.9	24.5	36.9	45.9	53.2	63.1	71.2	82.0	91.0	97.0	100	100
Processed	89.5	98.8	100	100	100	100	100	100	100	100	100	100	100	100

Metabolites

CV%	<5	<10	<15	<20	<25	<30	<35	<40	<50	<60	<70	<80	<90	<100
Raw	3.1	21.8	39.6	46.7	59.6	66.2	74.2	80.0	87.6	93.3	97.3	97.8	97.8	97.8
Corrected	25.8	43.6	63.1	76.0	82.2	90.2	93.3	96.9	99.1	99.5	100	100	100	100
Processed	96.3	100	100	100	100	100	100	100	100	100	100	100	100	100

To assess the potential existence of technical confounders in our lipidomics and metabolomics studies we calculated the coefficient of variation (CV) for the pooled quality control samples at each preprocessing step. After processing, 100 percent of the metabolites and 98.8 percent of the lipids had a CV of less than 10 percent.

Table S4: List of input molecules for each stage of systems analysis

Pathway Analysis using MetaboAnalyst Metabolites (n=51)	Pathway Analysis using LIPEA lipids (n=26)	OBaNK Interaction Analysis Multi-omics (n=109; l=26; m=51; p=32)	SVM and Cluster Analysis (n=25; l=8; m=15; p=2)
1-Methyladenosine	PC(36:7e)	PC(36:7e)	PC(36:7e)
1,3-diphopshateglycerate	PE(16:1e)(22:6)	PE(16:1e)(22:6)	PE(18:0)(20:3)
2-deoxyglucose-6-phosphate	BiotinylPE(32:5)	BiotinylPE(32:5)	CerP(d36:3+O)
2-hydroxygluterate	PIP2(31:6e)	PIP2(31:6e)	PE(16:1e)(20:4)
2-oxo-4-methylthiobutanoate	DG(8:0)(12:2)	DG(8:0)(12:2)	SM(d38:2)
2,3-Diphosphoglyceric acid	DG(4:0)(10:3)	DG(4:0)(10:3)	PC(32:1e)
3-hydroxybuterate	DG(4:0)(11:3)	DG(4:0)(11:3)	PC(32:0e)
3-methylphenylacetic acid	PC(17:0)(14:1)	PC(17:0)(14:1)	PC(35:1)
7-methylguanosine	PC(16:1e)(18:1)	PC(16:1e)(18:1)	deoxyadenosine
acadesine	CL(15:0)(18:1)(15:0)(18:1)	CL(15:0)(18:1)(15:0)(18:1)	shikimate
Acetyllysine	PE(40:5e)	PE(40:5e)	indoleacrylic acid
alanine	DG(6:0)(11:3)	DG(6:0)(11:3)	S-adenosyl-L-
anthranilate	ChE(20:4)	ChE(20:4)	homocysteine
arginine	DG(18:3e)	DG(18:3e)	1,3-diphopshateglycerate
asparagine	PI(18:0)(20:4)	PI(18:0)(20:4)	methylnicotinamide
aspartate	TG(16:0)(16:0)(17:0)	TG(16:0)(16:0)(17:0)	anthranilate
Carbamoyl phosphate	TG(6:0)(6:0)(14:1)	TG(6:0)(6:0)(14:1)	N-acetyl spermidine
D-gluconate	TG(12:1e)(6:0)(6:0)	TG(12:1e)(6:0)(6:0)	glucose-6-phosphate
D-glucono-delta-lactone-6-	PE(18:0)(22:6)	PE(18:0)(22:6)	hydroxyphenylacetic acid
phosphate	CerP(d36:3+O)	CerP(d36:3+O)	7-methylguanosine
D-sedoheptulose-1-7-phosphate	PE(16:1e)(20:4)	PE(16:1e)(20:4)	S-ribosyl-L-homocysteine
deoxyadenosine	PC(32:0e)	PC(32:0e)	fructose-6-phosphate
deoxyinosine	PC(32:1e)	PC(32:1e)	2,3-Diphosphoglyceric
deoxyribose-phosphate	PE(18:0)(20:3)	PE(18:0)(20:3)	acid
dGMP	SM(d38:2)	SM(d38:2)	N-Acetylputrescine
dihydroxy-acetone-phosphate	PC(35:1)	PC(35:1)	ACAN
dTTP		1-Methyladenosine	CFL1
fructose-6-phosphate		1,3-diphopshateglycerate	
glucose-6-phosphate		2-deoxyglucose-6-phosphate	
guanine		2-hydroxygluterate	
homoserine		2-oxo-4-	
hydroxyphenylacetic acid		methylthiobutanoate	
Indoleacrylic acid		2,3-Diphosphoglyceric acid	
Kynurenine		3-hydroxybuterate	
Methylmalonic acid		3-methylphenylacetic acid	

methylnicotinamide<br>N-acetyl spermidine<br>N-acetyl-glutamate<br>N-Acetylputrescine<br>N6-Acetyl-L-lysine<br>Nicotinamide Riboside<br>ornithine<br>Phenylpropiolic acid<br>ribose-phosphate<br>S-adenosyl-L-homocysteine-nega<br>S-ribosyl-L-homocysteine<br>SBP<br>shikimate<br>succinate<br>trehalose-sucrose<br>uracil<br>uridine		7-methylguanosine<br>acadesine<br>Acetyllysine<br>alanine<br>anthranilate<br>arginine<br>asparagine<br>aspartate<br>Carbamoyl phosphate<br>D-gluconate<br>D-glucono-delta-lactone-6-phosphate<br>D-sedoheptulose-1-7-phosphate<br>deoxyadenosine<br>deoxyinosine<br>deoxyribose-phosphate<br>dGMP<br>dihydroxy-acetone-phosphate<br>dTTP<br>fructose-6-phosphate<br>glucose-6-phosphate<br>guanine<br>homoserine<br>Hydroxyphenylacetic acid<br>Indoleacrylic acid<br>Kynurenine<br>Methylmalonic acid<br>methylnicotinamide<br>N-acetyl spermidine<br>N-acetyl-glutamate<br>N-Acetylputrescine<br>N6-Acetyl-L-lysine<br>Nicotinamide Riboside<br>ornithine<br>Phenylpropiolic acid<br>ribose-phosphate<br>S-adenosyl-L-homocysteine-nega<br>S-ribosyl-L-homocysteine	

		SBP<br>shikimate<br>succinate<br>trehalose-sucrose<br>uracil<br>uridine<br>ACAN<br>CFL1<br>CXCL11<br>H2AFZ<br>MUC1<br>NAMPT<br>INS<br>CD97<br>ICOS<br>PARK7<br>FAM107B<br>CD38<br>NGF<br>PPIF<br>THPO<br>DCN<br>MICA<br>HAPLN1<br>PRKCA<br>PTPN6<br>IGFBP2<br>FSTL1<br>CTSD<br>PROC<br>CCL28<br>CHRDL1<br>MSN<br>MMP14<br>CCL2<br>CXCL6<br>GNLY<br>CTSV	

Table S5: Functional analysis of lipids at the system level using LIPEA. The eighteen pathways included at least one input lipid.

Pathway name	Total	Hits	Lipids
Sphingolipid signaling pathway	9	1	Sphingomyelin
Linoleic acid metabolism	25	1	Phosphatidylcholine
alpha-Linolenic acid metabolism	23	1	Phosphatidylcholine
Autophagy - animal	4	2	Phosphatidylinositol, **Phosphatidylethanolamine**
Glycerophospholipid metabolism	26	4	Phosphatidylinositol, Cardiolipin, Phosphatidylcholine, **Phosphatidylethanolamine**
Glycosylphosphatidylinositol (GPI)-anchor biosynthesis	3	2	Phosphatidylinositol, **Phosphatidylethanolamine**
Necroptosis	4	1	Sphingomyelin
Sphingolipid metabolism	21	1	Sphingomyelin
Arachidonic acid metabolism	75	1	Phosphatidylcholine
Phosphatidylinositol signaling system	11	1	Phosphatidylinositol
Autophagy - other	3	2	Phosphatidylinositol, **Phosphatidylethanolamine**
Inositol phosphate metabolism	9	1	Phosphatidylinositol
Ferroptosis	11	2	1-Octadecanoyl-2-(5Z,8Z,11Z,14Z-eicosatetraenoyl)-sn-glycero-3-**phosphoethanolamine**, 1-Octadecanoyl-2-(7Z,10Z,13Z,16Z-docosatetraenoyl)-sn-glycero-3-**phosphoethanolamine**
Pathogenic Escherichia coli infection	1	1	**Phosphatidylethanolamine**
Tuberculosis	5	1	Phosphatidylinositol
Kaposi's sarcoma-associated herpesvirus infection	3	1	**Phosphatidylethanolamine**
Retrograde endocannabinoid signaling	8	2	Phosphatidylcholine
Choline metabolism in cancer	5	1	Phosphatidylcholine

Total: number of lipids in the pathway. Hits: lipids significantly different between delirium and control groups that are in the pathway. Lipids: Main lipid category and KEGG identifier returned by LIPEA.

Table S6: Leave-one-out-cross-validation (L1OXV) accuracy and area under curve (AUC) of the receiver's operating curve (ROC) for eight data sets used for regularized logistic regression with the elastic net analysis. AUC_L and AUC_U represent the lower and upper bounds for the 95% confidence interval for AUC. We applied the logistic regression with elastic net analysis on 8 data sets: (i) 26 lipids and (ii) 51 metabolites found to be significantly associated with delirium in this study, (iii) 32 proteins that are identified in Dillon et al., (iv) combination of these 3 sets (109 molecules), and 1.5 tFC cut-off applied to the sets (i-iii), which rendered (v) 8 lipids, (vi) 15 metabolites, (vii) 2 proteins, and (viii) 25 molecules that is the combination of (v-vii). For each data set, we calculated leave-one-out-cross-validation accuracy and the AUC of ROC.

	**L1OXV**	**AUC**	**AUC_L**	**AUC_U**
**25 Molecules**	**73.33**	**0.8622**	**0.6303**	**0.9593**
**8 Lipids**	43.33	0.5733	0.2858	0.7418
**15 Metabolites**	76.67	0.7911	0.5269	0.9353
**2 Proteins**	26.67	0.3733	0.1603	0.5976
**109 Molecules**	70	0.8089	0.5846	0.9286
**26 Lipids**	63.33	0.6933	0.4465	0.866
**51 Metabolites**	70	0.7067	0.4696	0.8707
**32 Proteins**	56.67	0.7244	0.5028	0.8839

Table S7: Metabolites common between plasma and CSF.

Comparison of metabolites that are associated with delirium based on (a) HiPOR CSF analysis at PREOP, (b) SAGES plasma analysis at PREOP, (c) SAGES plasma analysis at POD2. Metabolites common to all three are denoted in yellow, common in POD2 plasma and CSF denoted in grey, and common to PREOP plasma and CSF in green.

(a) CSF Preoperative (PREOP)

Metabolites	Nom-p	tFC
**D-gluconate**	0.0481	1.26
**S-ribosyl-L-homocysteine**	0.0069	1.57
**1-methyladenosine**	0.0128	1.40
**Acetyllysine**	0.0345	1.25
**Deoxyinosine**	0.0481	1.25
**D-glucono-Δ-lactone-6-phosphate**	0.0481	1.48
**D-sedoheptulose-1-7-phosphate**	0.0182	1.18
**Isocitrate**	0.0481	1.14
**S-adenosyl-L-homocysteine**	0.0005	1.95
**DL-pipecolic acid**	0.0481	1.10
**N-acetyl-glutamate**	0.0007	1.24
**Nicotinamide riboside**	0.0385	1.46
**Uracil**	0.0075	1.41

(b) Plasma Preoperative (PREOP)

Metabolites	Avg-p	tFC
**D-gluconate**	0.03	0.92
**S-ribosyl-L-homocysteine**	0.01	-0.86
**DL-pipecolic acid**	0.03	-0.94
**N-acetyl-glutamate**	0.02	-1.03
**Nicotinamide riboside**	0.07	0.74
**Uracil**	4.2E-03	-1.32

(c) Plasma Postoperative Day 2 (POD2)

Metabolites	Avg-p	tFC
**D-gluconate**	0.01	0.99
**S-ribosyl-L-homocysteine**	0.03	1.11
**1-methyladenosine**	0.01	1.55
**Acetyllysine**	0.02	1.54
**Deoxyinosine**	1.4E-03	1.90
**D-glucono-Δ-lactone-6-phosphate**	0.03	0.81
**D-sedoheptulose-1-7-phosphate**	0.03	1.06
**Isocitrate**	2.1E-03	1.52
**S-adenosyl-L-homocysteine**	0.03	1.02

Table S8: Quality Control Measures Applied to Instrumentation

Lipidomics	Metabolomics
The platform identifies an average of ~1500 unique lipid species per sample though this number changes based on sample quantity, complexity and purity. The identification are based not only on masses alone but also on MS/MS spectra that are searched against the LipidMaps and LipidSearch internal software databases. The spectra are graded with a letter designation and a mScore. Only scores above 5.0 are accepted and only grades A-C are accepted as these have fragment ions that match the fatty acid chains, head groups and various backbone for each lipid class. In addition, only precursor masses and fragment masses less than 8 ppm to the identified lipid are accepted. In most cases, the mass accuracy match is less than 2 ppm. A set of lipid standards that cover 5 different lipid classes are run on a daily basis for platform accuracy and quality control.	The targeted metabolomics platform used selected reaction monitoring (SRM) with positive/negative polarity switching to target 305 polar metabolites. Q3 fragment ion peak areas are calculated for identification and quantification. In short, the mass for each metabolite of interest is first filtered in Q1 by intact mass and then a specific and unique fragment ion is further filtered in Q3 after being fragmented in Q2. This method has very high specificity as all compounds were compared to known standards and chromatographic retention time is also used for identification accuracy. The end result is not a spectrum in the traditional sense but just a single fragment peak representing the analyte of interest. A cell extract that contains al targeted metabolites is run daily to assure platform accuracy and quality control.
